# Fixed Point Attractor Theory Bridges Structure and Function in *C. elegans* Neuronal Network

**DOI:** 10.3389/fnins.2022.808824

**Published:** 2022-04-25

**Authors:** Jian Liu, Wenbo Lu, Ye Yuan, Kuankuan Xin, Peng Zhao, Xiao Gu, Asif Raza, Hong Huo, Zhaoyu Li, Tao Fang

**Affiliations:** ^1^Department of Automation, Shanghai Jiao Tong University, Shanghai, China; ^2^Key Laboratory of System Control and Information Processing, Ministry of Education, Shanghai, China; ^3^Queensland Brain Institute, The University of Queensland, Brisbane, QLD, Australia

**Keywords:** fixed point, attractor, *C. elegans*, neural network, structure-function relationship

## Abstract

Understanding the structure–function relationship in a neuronal network is one of the major challenges in neuroscience research. Despite increasing researches at circuit connectivity and neural network structure, their structure-based biological interpretability remains unclear. Based on the attractor theory, here we develop an analytical framework that links neural circuit structures and their functions together through fixed point attractor in *Caenorhabditis elegans*. In this framework, we successfully established the structural condition for the emergence of multiple fixed points in *C. elegans* connectome. Then we construct a finite state machine to explain how functions related to bistable phenomena at the neural activity and behavioral levels are encoded. By applying the proposed framework to the command circuit in *C. elegans*, we provide a circuit level interpretation for the forward-reverse switching behaviors. Interestingly, network properties of the command circuit and first layer amphid interneuron circuit can also be inferred from their functions in this framework. Our research indicates the reliability of the fixed point attractor bridging circuit structure and functions, suggesting its potential applicability to more complex neuronal circuits in other species.

## Introduction

How neural network integrates information and encodes diverse behaviors is one of the key questions in neuroscience (Milo et al., 2002; Chen et al., 2006; Honey et al., 2010; Hu et al., 2016; Avena-Koenigsberger et al., 2017; Karla and Caridad, 2018; Schafer, 2018; Curto and Morrison, 2019; Lynn and Bassett, 2019; Morone and Makse, 2019). As an organism with a small nervous systems (Gray et al., 2005) whose neural connectivity has been completely mapped out, *Caenorhabditis elegans* provides an ideal model to address this question. However, due to the lack of knowledge in structure-function relationship, our understanding of how this relatively simple connectome generates diverse complex behaviors is far from complete (Hendricks et al., 2013) even three decades after the completion of the connectome (Sengupta and Samuel, 2009; Zhen and Aravinthan, 2015; Morone and Makse, 2019). To date, there have been two main approaches to investigate the relationship between structure and function. The first involves experimental research paradigms, which examine the behavioral activities of monitored organisms in the background of neuron manipulations such as targeted neuron optogenetics (Leifer et al., 2011) or laser ablation (Avery and Horvitzt, 1989; Chung et al., 2006), or analyzes the dynamic characteristics of neural activities by combining with optogenetics (Leifer et al., 2011) or laser ablation (Avery and Horvitzt, 1989; Chung et al., 2006). Following these research paradigms, researchers have identified circuits involved in different functions such as forward, backward movements in *C. elegans* (Chalfie et al., 1985; Gray et al., 2005; Kawano et al., 2011; Kato et al., 2015). The other approach is the theoretical research paradigm (Breakspear, 2017) which regards neural circuits as complex linear or non-linear neural networks (Liu et al., 2011; Yan et al., 2017; Kao and Hennequin, 2019). Early studies like White et al. (1986) used laser ablation method to provide the exact influence and function of certain neurons, revealing the important role of AVA and AVB. Wicks et al. (1996) additionally showed that neurons like PVC, who do not have obvious behavior during normal locomotion, are also influential. Rakowski et al. (2013) further focused on the command circuit of *C. elegans* and Haspel et al. (2010) the motor neuron, providing more detailed functional results of circuit and neurons. Furthermore, different theories such as control theory (Yan et al., 2017; Kao and Hennequin, 2019), complex network theory (Liu et al., 2011) and graph theory (Bullmore and Sporns, 2009; Liu et al., 2011; Deletoile and Adeli, 2017) are employed to study functional features such as dynamic characteristics and controllability of the neural circuit. These research paradigms have made respective advances in dissecting neural circuit functions (Yan et al., 2017). However, to date, findings in linking structure and function with distinguishing dynamic characteristics is still limited.

As a typical kind of attractor, the fixed point attractor of a system is an important dynamic characteristic that can maintain its steady states. It is well known that attractors have been applied to characterize the dynamic patterns of non-linear systems (Inagaki et al., 2019). In the complex biological nervous system, various aspects in various organisms, such as the posture dynamics of *C. elegans* (Rolls and Webb, 2012; Kunert et al., 2015; Nichols et al., 2017; Costa et al., 2018), the rhythmic locomotion in Aplysia (Bruno et al., 2017), and even complicated cortical information (Balaguer-Ballester, 2010; Finkelstein et al., 2021), are examined using this attractor theory. Interestingly, membrane potential in some C. *elegans* neurons such as AVAL/R (left and right AVA neurons, the same below), AVBL/R, AVEL/R and RIML/R are consistently bistable over time (Roberts et al., 2016), which implies a potential application of fixed point attractors to interpret these bistable phenomena. However, rarely has this theory been used to study *C. elegans* connectome and functions relationship.

In this work, we built a non-linear neural network model in *C. elegans*. Based on this model, we scanned *C. elegans* connectome and identified a rich repertoire of microcircuits with fixed point attractors. Given that the fixed point attractor of neural system represents neural circuit state, we select microcircuits that are essential to *C. elegans* motor movement and test their structure-function relationship. By constructing an attractor-based state machine and applying the attractor model on command circuit, we analyze the three fixed point attractors of the circuit, which corresponds to three different activity states within the circuit and three behavioral states. Laser ablation of specific neurons confirmed that the fixed point attractors from the connectome indicate circuit function and behavioral states. In addition, the fixed point attractor model can also implicate structure information from circuit and behavioral functions. When circuit and behavioral states are input into the model, the structural connectivity can be generated. In both cases, our work indicates important application of the fixed point attractor model in bridging neural network structure with its function.

## Analytical Framework for Structure-Function Relationship

### Non-linear Neuronal Network Model

To apply the attractor theory to study *C. elegans* neural network functions, we first establish a non-linear dynamic model for *C. elegans* neurons. Generally speaking, a biological nervous system is a non-linear dynamic system A major problem in describing a non-linear dynamic system such as *C. elegans* neural network using mathematically tractable linear controllability theory is the loss of biological interpretability (Jiang and Lai, 2019). One solution to obtain results with biological interpretability is to describe neural activity using non-linear neural dynamic models (Curto and Morrison, 2019). In this research, we described the dynamic of membrane potential change in *C. elegans* interneurons or motor neurons as follows (Izquierdo and Beer, 2013):


(1) $$\tau_{i}\,\frac{d\,x_{i}}{d\,t}=-x_{i}+\sum_{j=1}^{N}w_{j\,i}\,\sigma \,\left(x_{j}-\theta_{j}\right)+\sum_{l=1}^{N}g_{l\,i}\,\left(x_{l}-x_{i}\right)+b_{i}\,I_{i}$$

where *x_i_* represents the membrane potential relative to the resting potential; τ_*i*_ is the time constant; $\sum_{j=1}^{N}w_{j\,i}\,\sigma \,\left(x_{j}-\theta_{j}\right)$is the input from chemical synapses; $\theta_{j}$ represents the threshold, and *w*_*ji*_ the strength of chemical synapses between neurons *i* and *j*; *N* represents the total number of neurons; $\sum_{l=1}^{N}g_{l\,i}\,\left(x_{l}-x_{i}\right)$ is the input from gap junctions; with*g*_*li*_ being the conduct conductance between neuron *i* and neuron *j*; *b*_*i*_*I*_*i*_is the external stimuli; The Sigmoidal function σ(*x*) = 1/(1 + *e*^−*kx*^) represents the chemical synaptic potential of the neuron, where *k* regulates the transition rate of the sigmoidal function. The non-linear sigmoidal function in equation (1) makes it a better model to simulate the non-linear dynamics of a single neuron than the linear model in a physically or biologically interpretation way.

To study dynamic properties of a multi-neuron circuit, the non-linear dynamics of neuronal networks can be described by


(2) $$\dot{x}=\frac{1}{\tau }\,\left(G-L-E_{n\times n}\right)\,x+\frac{1}{\tau }\,W\,\sigma \,\left(x-\theta \right)+\frac{1}{\tau }\,B\,u$$

where *n* is the number of neurons within the circuit $x={\left[x_{1},\;x_{2\;},...,\;x_{n}\right]}^{T}$ represents the membrane potentials of *n* neurons at time *t*. σ(*x*−θ) = [σ(*x*_1_−θ),σ(*x*_2_−θ),…,σ(*x*_*n*_−θ)]^*T*^ implies that the non-linearity in Equation (2); *W* = $\left[\begin{array}{cccc} w_{11} & w_{21} & \cdots & w_{n1}\\ w_{12} & w_{22} & \cdots & w_{n2}\\ \vdots & \vdots & \ddots & \vdots \\ w_{1n} & w_{2n} & \cdots & w_{nn} \end{array}\right]$ represents the chemical synaptic connection matrix; *G* = $\left[\begin{array}{cccc} g_{11} & g_{21} & \cdots & g_{n1}\\ g_{12} & g_{22} & \cdots & g_{n2}\\ \vdots & \vdots & \ddots & \vdots \\ g_{1n} & g_{2n} & \cdots & g_{nn} \end{array}\right]$ represents gap junction connection matrix; $L=d\,i\,a\,g\,\left(\sum_{k=1}^{n}g_{k\,1},\sum_{k=1}^{n}g_{k\,2},\ldots ,\sum_{k=1}^{n}g_{k\,n}\right)$; *E*_*n*×*n*_*isaunitmatrix*; *u* represents external stimuli; *B* represents the input matrix. For simplicity, each neuron has the same time constant τ and threshold θ.

### Fixed Points of *C. elegans* Neural Circuit

The fixed point attractor within a neural circuit drives membrane potentials of different neurons within the circuit to stable values and maintains at this state for a relatively long time until switching to other stable states. These circuit level stable states eventually determine different behavioral states. According to the attractor theory, a fixed point is an equilibrium state that a system tends to reach and maintain regardless of external inputs. The fixed point attractors of *C. elegans* neuronal circuit are the solutions to the equation below


(3) $$\frac{1}{\tau }\,\left(G-L-E_{n\times n}\right)\,x+\frac{1}{\tau }\,W\,\sigma \,\left(x-\theta \right)=0$$

**FIGURE 1:**
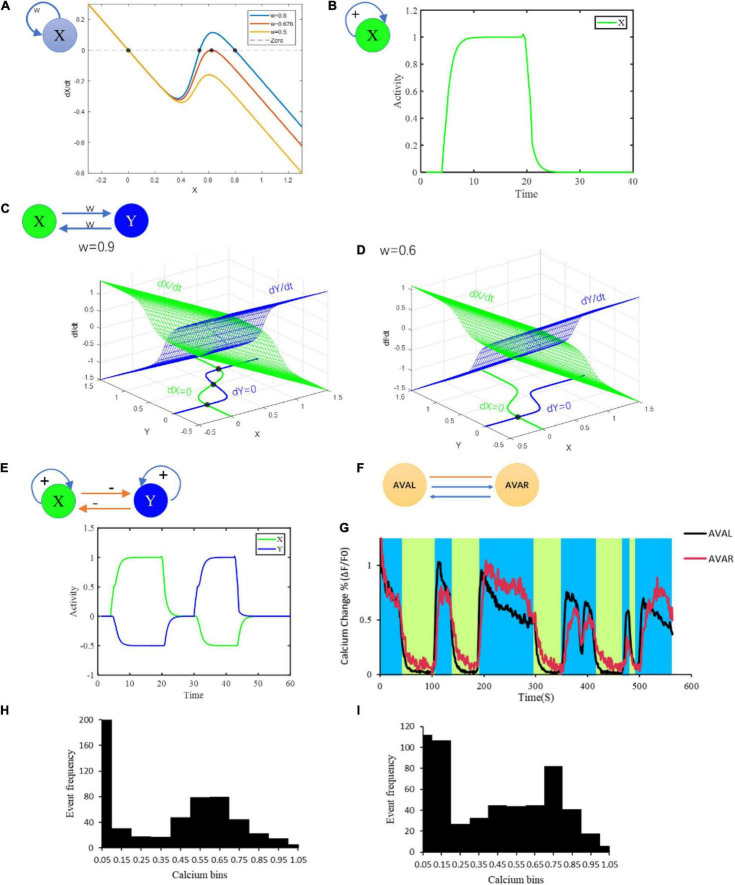
Multiple fixed points generated from different microcircuits. **(A)** Bifurcation analysis of at most three fixed points of neurons with at least one chemical synaptic self-loop. **(B)** Bistable activities of a neuron with self-loop. **(C)** Combined multistability of a complicated circuit composed of two simple motifs inhibiting each other in **(B)**. **(D)** Bifurcation analysis of a motif on the condition of strong connection and weak connection. **(E)** Attraction domains of three fixed points. Red dots represent the fixed points. The lines represent the state trajectories. *w* means connection strength; + and –represent excitatory and inhibitory connections, respectively. **(F)** Connection structure of AVAL and AVAR. **(G)** The neural activity of AVAL, AVAR changes with time. The blue area represents the period when AVAL, AVAR was in high neuro activity, and the light green area represents the period when AVAL and AVAR were in low neuro activity. **(H)** The distribution of AVAL’s neural activity changes. Its value was normalized. Bin size = 0.1. **(I)** The distribution of AVBL’s neural activity changes. Its value was normalized. Bin size = 0.1. Structural potential for multiple fixed points.

The fixed points of this system are the relative membrane potential vector. Taking a simple one-neuron circuit as an example, the number of fixed points increases from 1 to 3 with the increase of connection strength *w* (Figure 1A). However, due to the rapid rising phase in the sigmoidal function σ, the intermediate fixed point is an unstable fixed point and is almost impossible to be stably maintained. This is probably why neurons such as AVAL/R, AVBL/R, AVEL/R and RIML/R typically perform bistable states instead of tristable states. Our model also suggests a typical bistable pattern for single neuron under a two-fixed-points condition (Figure 1B). We also test a more complex circuit that includes two neurons interlinked by excitatory chemical synapses with the number of fixed points ranging from 1 to 3 (Figure 1D). Given a three-fixed-points condition, this circuit can maintain at three different states (Figure 1C). Notably, the fixed points of circuit are mainly determined by the connection matrices *G* and *W*. However, when the threshold θ is large enough, σ(*x*−θ)|_*x* = 0_ ≈0, then there must be a fixed point attractor of *x* = 0 which is independent of *G* and *W* (Figures 1A,D). This fixed point explains a resting state with neural activity closed to its resting potential.

Stable fixed points have corresponding attraction domains, in which any point along a specific trajectory tends to reach the particular fixed point (Figure 1E). This means that as long as the state is in the domain when external input terminates, the state of the system will be attracted to the corresponding attractor (Figure 1E). This enables the system to tolerate error and maintain at different stable states. However, in a more complex higher dimensional system, most fixed points are likely to be saddle points with stable and unstable manifolds (Vitolo et al., 2010). Unlike stable fixed points, unstable fixed points have no attraction domains, making them unlikely to be maintained.

To answer the question whether a neural circuit generates fixed point attractors, we present a sufficiency theorem for the emergence of multiple fixed points.

#### Sufficiency Theorem for Motifs With Multiple Fixed Points

In a motif composed of *n*(*n*≥1) neurons, there are unidirectional chemical synapses and/or symmetric bidirectional gap junctions among them. The edge weights of chemical synapses are positive or negative, with positive values indicating excitation and negative values indicating inhibition. In contrast, the weights of gap junctions are always positive. If the gradient potential in Equation (1) is used to describe the membrane potential of each neuron in the motif, the sufficiency condition for this motif to have multiple fixed points is that (i) there is at least one positive feedback loop dominated by excitatory chemical synapses, and (ii) the connection weights are properly adjusted without changing the chemical/electrical and excitatory/inhibitory properties of the synapses.

The Proof of this theorem is in Supplementary Information IV. It should be noted that the model is non-linear, which rises the computational difficulties. Some part of the proof is a qualitative justification instead of a strict mathematical proof. We hope that future mathematical work and non-linear results can perfect the theorem. As We can see from this theorem that, the positive feedback loop (Brandman and Meyer, 2008; Munteanu et al., 2014) dominated by excitatory chemical synapses plays a crucial role in the existing of multiple fixed points of neuronal networks. Therefore, the bistable or multistable regimes occur in the circuit rely on positive feedback loops.

### Attractor-Based State Machine Mediates State Switch

**FIGURE 2:**
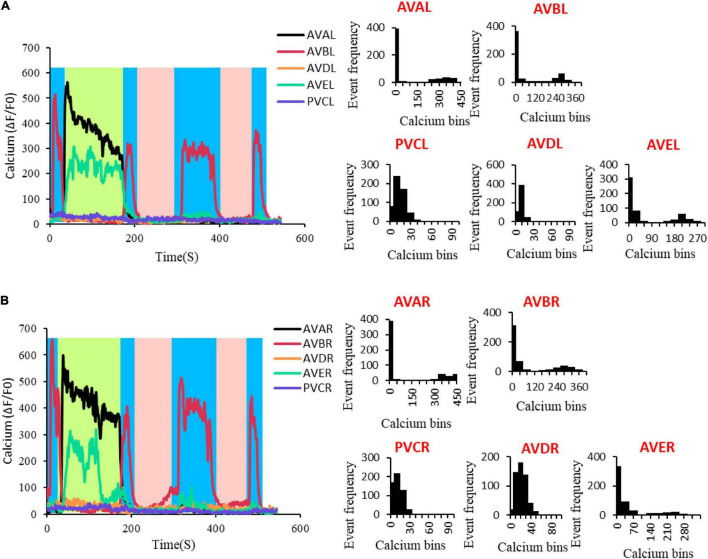
Multiple states in *C. elegans* neural activity. **(A)** The neural activity of AVAL, AVBL, AVDL, AVEL, and PVCL. The graph at the left of the panel is the neural activity changes with time, in which the blue area represents the period when AVBL was in high neuro activity, the light green area represents the period when AVAL and AVEL were in high neuro activity, and the pink area represents the period when all the neurons were in low neuro activity. The other demonstrated the distribution of the neural activity changes. AVAL, Bin size = 50; AVBL, Bin size = 40; AVDL, Bin size = 9; AVEL, Bin size = 30; PVCL, Bin size = 9. **(B)** The neural activity of AVAR, AVBR, AVDR, AVER, and PVCR. The graph at the left of the panel is the neural activity changes with time, in which the blue area represents the period when AVBL was in high neuro activity, the light green area represents the period when AVAL and AVEL were in high neuro activity, and the pink area represents the period when all the neurons were in low neuro activity. The other graphs demonstrated the distribution of the neural activity changes. AVAR, Bin size = 50; AVBR, Bin size = 40; AVDR, Bin size = 10; AVER, Bin size = 35; PVCR, Bin size = 8.

**FIGURE 3:**
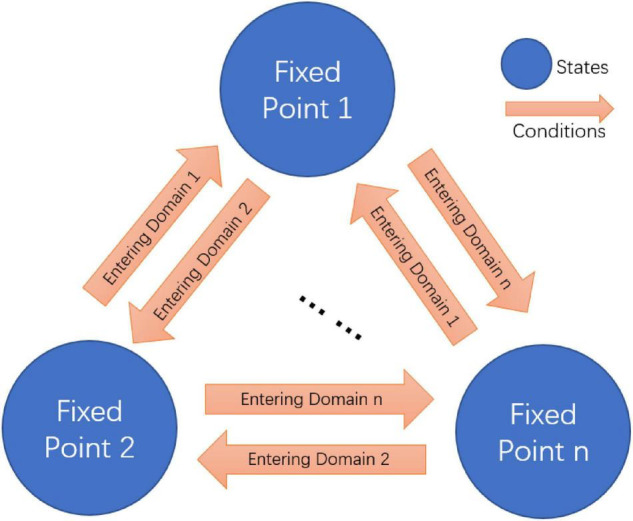
Fixed point attractors based finite state machine.

Activities in many *C. elegans* neurons can be switched between bistable states (Figures 2A,B). For example, AVA neuron can be switched from low calcium state to high calcium state, which leads to a behavioral state switching from forward movement to backward movement. To study how these neural circuit state and behavior state switch at the attractor level, we developed a useful tool of finite state machine (Beer, 2000; Buonomano and Maass, 2009; Helmstaedter, 2015) to describe this process. Distinct from a stochastic switch model with Markov mode (Roberts et al., 2016), we construct a state machine with multiple fixed point attractors as states and their attraction domains as switching conditions (Figure 3). Specifically, a neural network with *n* fixed points can switch among *n* states according to their corresponding attraction domains. When the system staying at one fixed point is disturbed by external inputs or internal noises, its state then drifts away from this fixed point (see Supplementary Information V). After the disturbance, if the system is still within the previous attraction domain, it then switches back to this fixed point. In contrast, once it reaches another attraction domain, the state then switches to the corresponding fixed point instead. This mechanism allows the system to reach preset states and switch among them accordingly.

Animals stay in a disturbed environment with drifting ambient temperature and other unpredictable variables. It is important for them not only to maintain stable neural network and behavioral states but also quickly switch to different states to adapt the change. If different fixed points drive different neural network and behavior functions, the state machine composed of multiple fixed points explains the switch among these different states. By combining the fixed point theory and the state machine, we can distinguish the function of neurons in terms of both states (multiple fixed points) and conditions (attraction domains). We find that some neurons are essential for the existence of multiple fixed points, while others may contribute to the formation of the corresponding attraction domains of these fixed points. A state machine constructed in this way relates non-linear dynamics characteristic of neuronal network to circuit function. It not only has important implications for our further understanding of the circuit function, but also provides us with the potential requirement of the non-linear dynamics for the realization of certain function, which is crucial for structure deduction.

### Structure-Function Analytical Paradigms

Since we have linked multiple fixed points characteristic to both structure and function, we are now able to develop an analytical framework to interpret structure-function relationship by using fixed point theory. The analytical framework consists of two research paradigms: *From Structure to Function* and *From Function to Structure*, as shown below.

**Table:** 

**Paradigm I:** From structure to function.

(1) Acquiring circuit connectome information from biological studies and its adjacency matrices.
(2). Describing the bistable circuit by non-linear model of Equation (2).
(3) Calculating the fixed points of the circuit according to Equation (3).
(4). Constructing a state machine by using the fixed points calculated and their corresponding attraction domains.
(5) Analyzing the network functions of neurons according to their contribution to corresponding fixed points or attraction domains.
(6) Conducting bio-experiments to verify the results.


Supported by different connectome information or neural activity data, this framework allows us to interpret functional principle from intricate circuit structure or deduct structure properties from characteristical neural activities to ultimately understand how the circuit works. We now apply this framework to neural circuits in *C. elegans* and obtain their functional interpretability and structural deduction reliability.

**Table:** 

**Paradigm II:** From function to structure.

(1) Acquiring neural activities of the circuit studied, especially those with bistability, from experimental observation
(2) Determining neuronal functions and fixed points of circuit performance through analyzing neural activities.
(3) Constructing a state machine by using the fixed points determined and their corresponding attraction domains.
(4) Deducing potential structural properties from the emergence of fixed points and corresponding attraction domains.
(5) Conducting bio-experiments and searching previous studies to verify the results.


## Structure-Function Interpretation

### Potential Network Motifs for Bistability

To gain insight into neural circuits function, we first conduct the study from the view of circuit motifs. A neural network could include a lot of distinct motifs with featured chemical/electrical connections. By screening *C. elegans* connectome (279 non-pharyngeal neurons), we find that the one-node motif in which a neuron connects to itself through chemical synapses appears 34 times, among which one-node motif that connect to itself through chemical and electrical synapses appears 3 times. In addition, the two-node motifs where two neurons connect to each other via chemical synapses alone or via both chemical and electrical synapses appear 252 and 93 times in the network, respectively (potential circuit motifs are shown in Supplementary Information_Motifs). Notably, these recurrent motifs have the potential for bistability, and are likely related to multiple fixed points. Therefore, these motifs may help us predict bistability related functional activities from the neuronal network structure. Actually, neurons within the above motifs, such as AIB, AVA, AVB, AVE, RIM, and CEP do show bistability activities. For an example, AVAL and AVAR are connected with each other with chemical synapses and gap junction as well. Together the two neurons form a motif (Figure 1F) with the potential for bistability. In a moving worm, activities of AVAL and AVAR do show distinguishing bistability (Figures 1G–I) that is crucial for locomotion. These results suggest the potential application of fixed point theory to interpreting and even predict bistability related function.

### Corresponding Neuronal Activities Encoded by Fixed Points

**FIGURE 4:**
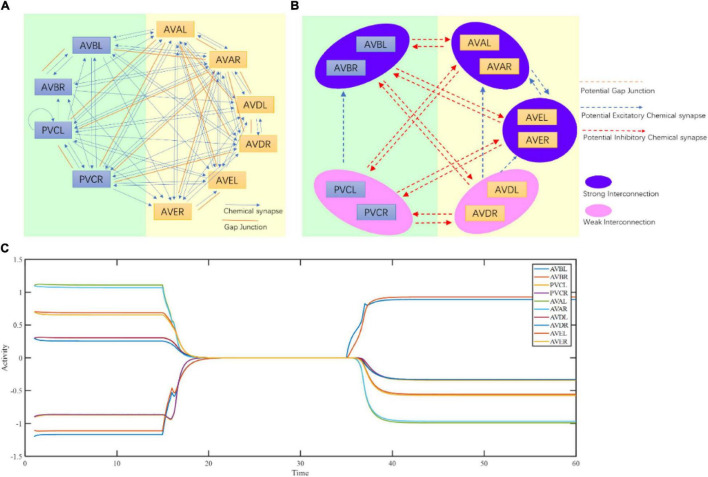
Command circuit of *C. elegans.* **(A)** Connectome of command circuit. **(B)** Implicit structure deduction of command circuit. **(C)** Three fixed points of the command circuit calculated by Equation (3).

To further test the ability of interpreting function of the proposed framework at the circuit level, we apply a structure-function research paradigm to the forward–backward switching circuit to interpret the states and functions of command interneurons. Previous research indicated that 10 left-right symmetrical neurons, namely AVBL/R, PVCL/R, AVAL/R, AVDL/R, and AVEL/R neurons, form a command circuit that plays an important role in the forward/reverse switch in *C. elegans* (Rakowski et al., 2013). Rich connectivity among these neurons have been discovered in the connectome Figure 4A. So far it has been generally difficult to obtain the actual weights of connections. Our main goal is to interpret its working principle rather than guessing its function. With the help of its known function, the original adjacency matrices regarding the number and size of synapese (Rakowski et al., 2013) (Supplementary Tables 1, 2) of the circuit are acquired and adjusted (see Section “Method,” Supplementary Tables 7, 8) for the computation of fixed points using Equation (3). During the process, adjustment strictly follow the guiding of the actual connectome of *C. elegans*, with only the weights of connection adjustable. The existence of chemical and electrical connections must remain unchanged. It should be noted that the exact excitatory or inhibitory property of certain synapses are still debatable. For an example Rakowski and Karbowski (2017) suggests neuron PVC generates negative current to neuron AVB. This contradicts to the experiment results that the activation of PVC and trigger the activity of AVB and other ablation experiments (Chalfie et al., 1985). Thus, we choose to believe the chemical connection from PVC to AVB is excitatory. Also, the inhibitory connections between interneurons mentioned in Fenyves et al. (2020) do not contradict to our assumptions of strong excitatory connections between the paired neurons, such as between AVBL and AVBR, and between AVAL and AVAR. From the weight matrices, three fixed points in total (Normal column of Table 1 and Figure 4C) are then acquired. The fixed point 1 corresponds to strong activations of AVAL/R and AVEL/R, mild activation of AVDL/R and strong inhibitions of AVBL/R and PVCL/R. The fixed point 2 corresponds to strong activation of AVBL/R, mild inhibition of PVCL/R and strong inhibitions of AVAL/R and AVEL/R. The fixed point 3 corresponds to no activations in any of the five pairs of neurons.

**TABLE 1:** Fixed point attractors of command circuit computed under different conditions.

Different conditions Neurons		Normal	AVA ablation	AVB ablation	AVD ablation	AVE ablation	PVC ablation	Gap removed	Gap AVA to PVC removed
AVBL	Fixed point 1	0.9	1	none	0.9	0.9	0.9	1	0.9
AVBR		0.9	1		0.9	0.9	0.9	1	0.9
PVCL		–0.3	0		–0.4	–0.4	0	0	0
PVCR		–0.3	0		–0.4	–0.4	0	0.1	0
AVAL		–1	0		–1.1	–1.3	–1.2	–2	–1.2
AVAR		–1	0		–1	–1.2	–1.2	–2	–1.2
AVDL		–0.3	–0.2		0	–0.4	–0.4	–0.2	–0.4
AVDR		–0.3	–0.2		0	–0.4	–0.4	–0.2	–0.3
AVEL		–0.6	–0.1		–0.6	0	–0.7	–0.1	–0.7
AVER		–0.6	–0.2		–0.6	0	–0.7	–0.2	–0.7
AVBL	Fixed point 2	–1.2	none	0	–1.2	None	–1.1	–1.5	–1.1
AVBR		–1.1		0	–1.1		–1.1	–1	–1.1
PVCL		–0.9		–0.8	–0.9		0	–2	–2
PVCR		–0.9		–0.8	–0.9		0	–2	–2
AVAL		1.1		1.2	1.2		3	1	1.9
AVAR		1.1		1.2	1.1		3	1	1.9
AVDL		0.3		0.3	0		0.7	0.1	0.4
AVDR		0.3		0.3	0		0.7	0.1	0.3
AVEL		0.7		0.7	0.7		1.7	0.2	1.1
AVER		0.7		0.7	0.7		1.7	0.2	1.1
AVBL	Fixed point 3	0	0	0	0	0	0	0	0
AVBR		0	0	0	0	0	0	0	0
PVCL		0	0	0	0	0	0	0	0
PVCR		0	0	0	0	0	0	0	0
AVAL		0	0	0	0	0	0	0	0
AVAR		0	0	0	0	0	0	0	0
AVDL		0	0	0	0	0	0	0	0
AVDR		0	0	0	0	0	0	0	0
AVEL		0	0	0	0	0	0	0	0
AVER		0	0	0	0	0	0	0	0

To verify these deductions, we record spontaneous calcium responses in these 10 neurons. The results showed that the calcium traces of AVAL/R, AVEL/R, and AVBL/R display typical two steady activity states that can be effectively maintained and switched between (Figures 2A,B and Supplementary Figures 5A,B). In addition, these ten neurons perform three stable states (left panel of Figures 2A,B). In state 1, AVAL/R and AVEL/R are activated and no activations in any other neurons, which corresponds to the fixed point 1 (light green region in left panels of Figures 2A,B). In state 2, AVBL/R are activated and no activations in any other neurons, which corresponds to the fixed point 2 (blue region in left panels of Figures 2A,B). In state 3, all the 10 neurons are not activated, which corresponds to the fixed point 3 (pink region in left panels of Figures 2A,B).

The activations of AVA and AVE lead to backward movements in worms (Kato et al., 2015), suggesting the fixed point 1 triggers reversal behavior. AVB neuron is inhibited during reversal. In contrast, the activation of AVB neuron leads to forward movements, indicating the fixed point 2 triggers forward behavior. AVA and AVE neurons are inhibited during forward locomotion. The state 3 corresponds to a sleeping state which none of these 10 neurons is activated (Cho and Sternberg, 2014; Nichols et al., 2017), suggesting the fixed point 3 is in a sleeping state. Notably, animals are commonly observed in state 1 and state 2, and less frequently in state 3. They either move forward or backward. However, worms in a molting state have high possibility of entering a sleeping state, which is the state 3. Indeed, most of the worms in a non-molting period only have two states without the state 3 (Supplementary Figures 5A,B). This can be explained by the size of attraction domain and whether the fixed point is a stable fixed point. The larger an attraction domain is, the more likely the state is to maintain at its corresponding fixed point. Studies on simpler structures (shown in Figure 1) suggest stronger chemical interconnections lead to larger attraction domain of the fixed point where the related neurons stay active. These suggest a simple rule that stronger internal chemical connections result in longer maintain for the fixed point where the corresponding neurons are active. In this 10-neuron circuit, attraction domains of the first two fixed points follow this rule. The more space the former two attractions domains take, the less is left for the third. The third fixed point, however, is more likely to be an unstable fixed point during moving, and has no attraction domain. Under the influence of noise, the state is unlikely to be maintained at an unstable fixed point. This is why lasting pausing state is rarely observed when a worm is active. But this particular unstable fixed point can become a stable fixed point during sleeping, ensuring the related neurons stay inactive until woken.

**FIGURE 5:**
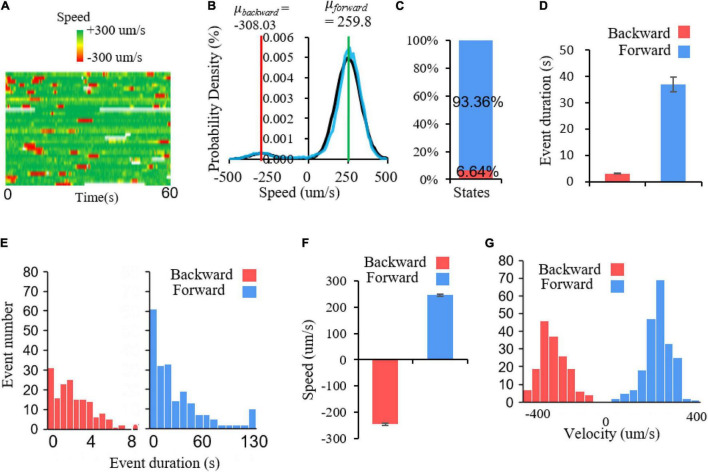
Multiple locomotory states in *C. elegans* movement. **(A)** Speed heat map of a group of worms. Each row represents a speed trace, green and red represent forward and backward speed, respectively. **(B)** Probability density of speed distribution. The bimodal Gaussian function $f\,\left(x\right)=\frac{1}{\delta \,\sqrt{2\,\pi }}\,e^{-\frac{1}{2}\,{\left(\frac{x-\mu }{\delta }\right)}^{2}}$is applied to fit *C. elegans* speed distribution. Black represents fitting curve and the blue represents raw speed distribution. The parameters for two models are: μ*_*forward*_* = 259.8, δ*_*forward*_* = 77.79164; μ*_*backward*_* = −308.03, δ*_*backward*_* = 62.79239. **(C)** The percentage of two locomotory states. Forward movement accounts for 93.36% of locomotory time and backward movement only account for 6.64%. **(D)** Average duration for forward and backward events. *T*-test, *p* < 0.001. **(E)** The duration distribution of backward and forward events. Backward events: bin size = 0.6; forward events: bin size = 10. **(F)** Average speed of forward and backward events. *N*_*forward*_ = 209, *N*_*backward*_ = 164. **(G)** The speed frequency distribution of forward and backward events. Bin size = 44.

To further verify it at the behavioral level, we record locomotory speed when animals are freely conducting local search in a new environment without food. In non-mouting worms, during the local search, their forward movements at a relatively high speed are interrupted by reversals (Figure 5A). The speed histogram indicates two Gaussian distributions in the positive and negative speed areas, with center values of +259.8 and −308.03 μm/s, respectively (Figure 5B). This suggests that there are two major states of C. *elegans* movement during local search, namely the forward movement and the backward movement, which correspond to fixed point 2 and 1, respectively. Notably, there is a small distribution around speed 0 (Figures 5A,B), which is a still state, corresponding to fixed point 3. This portion is too small and difficult to separate it from another two states. This implies that the corresponding fixed point is an unstable fixed point. The major two states are not evenly distributed. Forward movement accounts for 93.36% whereas backward movement accounted for only 6.64%, which indicates a strong forward state bias in local search behavior (Figure 5C). This also suggests that the sizes of distinct fixed point attraction domains could be very different, thus occupies different percentage of behavior states. To explore how this locomotory bias is maintained during movement, we further examine the single forward and backward events. The results show that the duration of single forward events is much longer than that of backward events, indicating that the forward state lasts much longer (Figures 5D,E). Although backward event is much brief, lasting only seconds, the absolute speed can be comparable to the forward events (Figure 5F). In particular, the speed frequencies of these two states are roughly evenly distributed, with opposite directions (Figure 5G). All these results indicated that there are multiple states in *C. elegans* locomotion, a brief backward state, a long-lasting forward state and probably a small portion of pause state. This pause state is further confirmed in molting worms. Spontaneous locomotion in sleepy animals show clearly three states, a backward movement state which corresponds to the fixed point 1, a forward movement state which corresponds to the fixed point 2, and a still state which corresponds to the fixed 3 (Supplementary Figures 3A–D).

These data verify the existence of multiple fixed points and confirms that these fixed points constrain the command circuit states. By switching among these multiple fixed points, the animal displays corresponding motor states such as reversal, forward movements and pause. All these results suggest that these fixed points could in principle reveal the behavioral patterns encoded by the command circuit.

### Neuronal Function of Constituting States

The maintenance of stable activity patterns of neurons is controlled by the dynamics converging toward the fixed points retained in the neuronal network structure, so that the neurons that affect the existence of fixed points should play a crucial role in the transition between different behavioral states. To identify neurons contributing to the maintenance of fixed points within the command circuit, we conducted a series of computations under different ablation conditions using Equation (3), with their influence on the presence of multiple fixed points in Table 1. The prediction shows that the ablation of either AVAL/R or AVEL/R leads to the impairment of fixed point 2, which is the state of backward behavior. Similarly, the ablation of AVBL/R results in the impairment of fixed point 1, which is the state of forward behavior. This implies that the ablation of AVAL/R, AVBL/R, or AVEL/R might have the greatest impact on behavior. However, the ablation of either AVDL/R or PVCL/R does not affect the existence of three fixed points. From our computational analysis, we can speculate that ablation of AVAL/R or AVEL/R will impair the backward movement, and the ablation of AVBL/R will attenuate the forward movement. However, the ablation of AVDL/R or PVCL/R tends to have relatively little effect on the forward–backward movement. These ablations are also in tune with previous ablations results in Rakowski et al. (2013) which concerns the average time length of locomotion states rather than the distribution.

It is also predicted through our theoretical computing that under the removal of all gap junctions, there are still three fixed points in the command circuit, in Table 1. This shows that the gap junctions have little influence on the existence of multiple fixed points. The gap junction between AVA and PVC neurons is particularly interesting because it links two competitive classes of neurons. We speculate that AVA neuron provides small potential stimulation to PVC through this gap junction when active, increasing the possibility of a forward switch.

**FIGURE 6:**
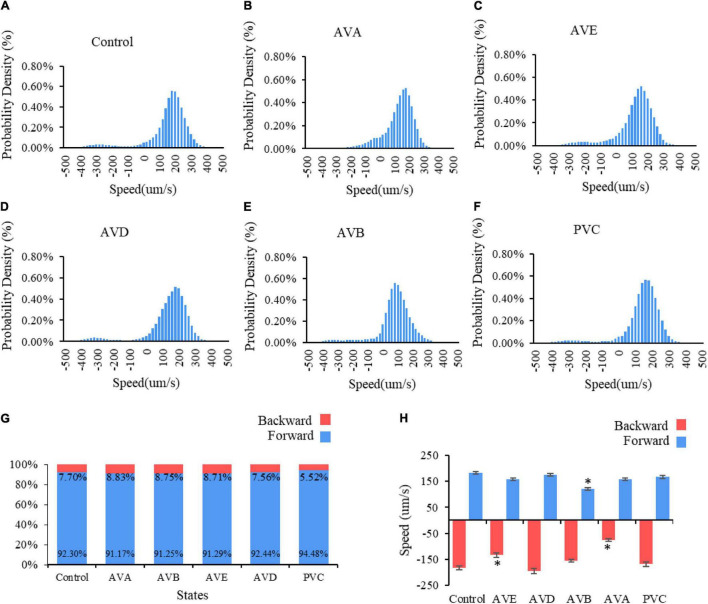
Two locomotory states in *C. elegans* movement: forward and backward. Probability density of speed distribution for *C. elegans* **(A)** without neuro impairment, bin size = 20; **(B)** with AVD impairment, bin size = 20; **(C)** with PVC impairment, bin size = 20; **(D)** with AVA impairment, bin size = 20; **(E)** with AVE impairment, bin size = 20. Panel **(F)** with AVB impairment, bin size = 19. **(G)** The percentage of two locomotory states. **(H)** Average velocity of two locomotory states. * means significantly different from control group.

To verify the theoretical predictions, we conducted laser ablation experiments to ablate these neurons pair by pair (shown in Figure 6). The results showed that when AVAL/R or AVEL/R were ablated, the reversal speed is significantly decreased, suggesting that the backward movement is impaired (Figures 6B,C,H). In contrast, when AVBL/R were ablated, the forward speed is significantly reduced, suggesting an impairment in forward movement (Figures 6E,H). However, ablations of PVCL/R and AVDL/R have mild effect on the forward–backward movement (Figures 6D,F,H). These results suggested that neurons AVBL/R, AVAL/R and AVEL/R are critical in generating multiple stable fixed points, while AVDL/R and PVDL/R are less important in this process, which supports our theoretical predictions.

### Neuronal Function of Constituting Switching Conditions

Since PVCL/R and AVDL/R do not contribute to the existence of multiple fixed points, we now further ponder what role they play in the forward and backward behaviors. As we have suggested, state transition depends on the change in the attraction domain in which the state is located. Whatever the initial state is, it must fall into one of the three fixed points for the effective state transition of the command circuit. Using the adjusted adjacency matrices, we try to observe how the command circuit transit from a range of initial states to the final state. From theoretical calculation results in Table 2, activation of either AVBL/R or PVCL/R leads to fixed point 1, while activation of AVDL/R, AVEL/R or AVDL/R leads to fixed point 2. In other words, at fixed point 1, only AVBL/R maintain stable activity, while at fixed point 2, AVAL/R and/or AVEL/R neurons maintain stable activity. From the view of non-linear dynamics, it can be inferred that although AVDL/R and PVCL/R are not active at fixed points, their existence enlarges the attraction domains, which will help the circuit to collect external input and respond correctly to the environment. It is consistent with animal experimental facts (Kato et al., 2015) that activation of AVBL/R does not activate PVCL/R, and that activation of AVAL/R leads to the activation of AVDL/R. Combined with the position information of AVDL/R in the head and that of PVCL/R in the tail, it is reasonable to deem that AVDL/R collects information from the anterior part to trigger the backward movement, and PVCL/R collects information from the posterior part to trigger the forward movement (Chalfie et al., 1985). Therefore, AVDL/R and PVCL/R may perform their functions by expanding attraction domains and constructing the switching conditions for the state machine.

**TABLE 2:** The state transition to different fixed points for activation of specific neuron pairs.

	Neurons	Activation or deactivation					
**Initial State**	AVBL	1	0	0	0	0	0
	AVBR	1	0	0	0	0	0
	PVCL	0	1	0	0	0	0
	PVCR	0	1	0	0	0	0
	AVAL	0	0	1	0	0	0
	AVAR	0	0	1	0	0	0
	AVDL	0	0	0	1	0	0
	AVDR	0	0	0	1	0	0
	AVEL	0	0	0	0	1	0
	AVER	0	0	0	0	1	0
Final state		Fixed point 1		Fixed point 2			Fixed point 3

## Function-Structure Deduction

### Command Circuit Structure Deduction

Connectome implies circuit functional information, which has been explored using our structural-to-functional research paradigm. Notably, the activity pattern of a circuit may implicitly contain its structural information (Real et al., 2017). To test this idea, we apply the functional-to-structural research paradigm to deduct command circuit connectivity. Using the calcium activity data in the previous section (Figure 2 and Supplementary Figure 5), we can obtain the functional activities of the neurons in this circuit that are involved in both forward and backward movement. We first divide the neurons in this circuit into two groups, namely two pairs of neurons (AVBL/R and PVCL/R) that promoted the forward behavior, and three pairs of neurons (AVAL/R, AVEL/R, and AVDL/R) that promoted the backward behavior, according to the different functions of neurons in this circuit for the forward and backward behaviors (Kato et al., 2015). During forward behavior, AVBL/R maintain relatively steady plateau potentials. Similarly, during backward behavior, AVAL/R and AVEL/R also maintain steady plateau potentials together. According to the attractor-based state machine we construct, the activity pattern of the circuit is the result of the switch between its multiple fixed points. Through the analysis of its calcium activity data, we found three steady states within this circuit, which implies three fixed points in this circuit. Specifically, fixed point 1 occurs to keep AVBL/R active when the worm moves forward, fixed point 2 occurs to ensure that AVAL/R and AVEL/R remain active when the worm moves backward, and fixed point 3 occurs when all the neurons were in low neuro activity. These three fixed points also occurs in sleeping worms (see Supplementary Information VII B) but with different durations. The state switching of this circuit mainly occurs between these two fixed points, according to the principle of the fixed-point-based state machine as shown in Figure 3. Then we analyze the neuronal function according to their contribution to the states (multiple fixed points) and switching conditions (attraction domains) for the state machine. As bistable neurons curtail for the emergence of multiple fixed points, AVAL/R, AVEL/R, and AVBL/R are involved in the state maintenance, whereas AVDL/R and PVCL/R aids in constituting the switching condition. With the help of our proposed theorem, for the purpose of ensuring the existence of multiple fixed points and corresponding attraction domains as analyzed in the previous section, we deduct the potential connections between these neurons as shown in Figure 4B. Some of the deduced results are in line with the experimental facts of *C. elegans* connectome, as shown in Table 3.

**TABLE 3:** Deduction of structure properties.

Conditions	Deduced results of command circuit	Experimental facts	Deduced results of first layer amphid interneuron circuit	Experimental facts
Two neurons need to be strongly connected to each other to form fixed points	AVBL and AVBR are strongly connected	Eight gap junctions between them, 3 chemical synapses from AVBL to AVBR, 1 chemical synapse from AVBR to AVBL	AIBL and AIBR are strongly connected	AIBL has 1 chemical synapse self-loop
	AVAL and AVAR are strongly connected	Eight gap junctions between them, 9 chemical synapses from AVAL to AVAR, 7 chemical synapses from AVAR to AVAL	AIYL and AIYR are strongly connected	One gap junction between them, 2 chemical synapses from AIYR to AIYL
	AVEL and AVER are strongly connected	One gap junction between them, 1 chemical synapses from AVER to AVEL		
Neurons without bistability do not strongly activate each other to form fixed points	AVDL and AVDR are weakly connected, or only strongly connected with gap junctions	Four gap junctions between them, 3 chemical synapses from AVDL to AVDR, 3 chemical synapses from AVDR to AVDL	AIAL and AIAR are weakly connected, or only strongly connected with gap junctions	AIAR is not connected to AIAL
	PVCL and PVCR are weakly connected, or only strongly connected with gap junctions	Twenty-seven gap junctions between them, 3 chemical synapses from PVCL to PVCR, 5 chemical synapses from PVCR to PVCL, 1 chemical synapse self-loop on PVCL	AIZL and AIZR are weakly connected, or only strongly connected with gap junctions	AIZL and AIZR are only connected with gap junctions
Neurons forming fixed points with opposite bistability tend to inhibit each other	AVBL/R inhibit AVAL/R or AVEL/R or both, and are inhibited by AVAL/R or AVEL/R or both.	AVB and AVA are strongly connected, and there are evidence showing that they inhibit each other (Chalfie et al., 1985; Roberts et al., 2016; Maluck et al., 2019).	AIBL/R and AIYL/R are most likely to interconnect with inhibitory chemical synapses	There are chemical synapses between AIB and AIY
Neurons forming attraction domains may either excite neurons forming the corresponding fixed points or inhibit those forming the opposite fixed points.	PVCL/R excite AVBL/R, or inhibit AVAL/R or AVEL/R, or do both	There are inhibitory chemical synapses from PVC to AVB and AVA (Chalfie et al., 1985; Maluck et al., 2019)	AIAL/R excite AIYL/R, or inhibit AIBL/R, or do both	There are rather many chemical synapses from AIA to AIB and proves to be inhibitory (Shinkai et al., 2011)
	AVDL/R excite AVAL/R or AVEL/R, or inhibit AVBL/R, or do both.	There are rather many chemical synapses from AVD to AVA	AIZL/R excite AIBL/R, or inhibit AIYL/R, or do both	There are rather many chemical synapses from AIZ to AIB
Neurons forming fixed points may inhibit neurons forming attraction domains of the opposite fixed points	AVBL/R inhibit AVDL/R	There are chemical synapses from AVB to AVD and AVA	AIYL/R inhibit AIZL/R	There are rather many chemical synapses from AIY to AIZ and proves to be inhibitory (Li et al., 2014)
	AVAL/R inhibit PVCL/R	There are inhibitory chemical synapses from AVA to PVC and AVA (Chalfie et al., 1985; Maluck et al., 2019)	AIBL/R inhibit AIAL/R	There is one chemical synapse from AIB to AIA
	AVEL/R inhibit PVCL/R	There are inhibitory chemical synapses from AVE to PVC and AVA (Chalfie et al., 1985; Maluck et al., 2019)		

### First Layer Amphid Interneuron Circuit Structure Deduction

We further test this paradigm by using the first layer amphid interneuron circuit of *C. elegans*. By comparing and analyzing the observed data for the calcium activity of each neuron in the first layer amphid interneuron circuit, we qualitatively obtain some specific functions and some specific dynamic properties of the neurons that make up this circuit. The analysis process is similar to that above, and the deduced structure properties are also shown in Table 3. We deduce the inhabitation between AIBL/R and AIYL/R, strongly interconnected pairs of AIBL/R and AIYL/R, weakly interconnected pairs of AIAL/R and AIZL/R, and some other inhibition and excitation connections within the circuit (Table 3). The deduced results are satisfactory because most of the deduced connections can be found existed in connectome data (Jarrell et al., 2012; Brittin et al., 2018; Cook et al., 2019) and previous studies (Chalfie et al., 1985; Shinkai et al., 2011; Li et al., 2014; Roberts et al., 2016; Maluck et al., 2019) (Table 3), proving the effectiveness of our proposed framework. In addition to these available convincing experimental facts, there are still some deduction results on the excretory/inhibitory and strength of the connection within these two circuits that need to be further supported by future biological experiments.

## Discussion and Conclusion

The development of a potentially powerful tool for the interpretation of connectome function may require our understanding of the relationships between neuronal network structure, dynamics, and function (Sankaraleengam et al., 2016), however, the establishment of these theoretical relationships remains a challenge (Curto and Morrison, 2019). At present, more research on the relationship between structure and function is to analyze the dynamic characteristics of the circuit from a large number of experimental observation data of neural activity (Inagaki et al., 2019), and further infer or explain its function (Sankaraleengam et al., 2016; Real et al., 2017). This means that, in these studies, intrinsic dynamic characteristics play a particularly critical role in bridging the relationship between structure and function. The structure of a neuronal network cannot decide its function alone (Honey et al., 2010). A certain structure constrains its internal dynamics to realize its function. Fixed point attractor can be used to explain functions associated with bistability, which is rather common in the nervous system of *C. elegans* (Kunert et al., 2015) and other animals (Kolbjrn et al., 2013). Some structures, even as simple as motifs, can result in multiple fixed points in a neuronal network. If fixed points are regarded as states, a finite states machine can be built to explain how neural circuits switch between stable states in which they perform their functions. Furthermore, the function of neurons in the circuit can be subdivided according to their roles in the state machine and their contribution to the fixed points. Our proposed analytical framework consists mainly of two processes. The first is that if enough structural properties are known, the principle of circuit function switching with respect to bistability or multistability is analyzed from the structure-to-function research paradigm; the second is that if sufficient functional and neuronal activity information is known, possible structure properties can be deduced from the function-to-structure research paradigm to provide guidance for anatomical experiments. The framework was tested by using the command circuit and the first layer amphid interneuron circuit of *C. elegans*.

In addition to the bistability of calcium activity, similar bistable potential was also found in electrophysiological experiments. Since the resulting bistability reflects the multiple fixed points that exist in the neuronal network, we have reasons to believe that the plateau potential with bistability in electrophysiology may also be explained by multiple fixed points. RMD neurons of *C. elegans*, for example, have two stable resting potentials, one near −70 mV and the other near −35 mV (Mellem et al., 2008; Lockery and Goodman, 2009). These RMD neurons consist of six neurons, namely RMDDL, RMDDR, RMDL, RMDR, RMDVL, and RMDVR. The adjacency matrices of RMD neurons are listed in Supplementary Tables 5, 6. RMDL and RMDR are strongly connected with each other through excitatory chemical synapses. This may be the reason why the plateau potential appears in electrophysiology of RMD neurons. Note that the electrophysiological experiments and the calcium activity experiments are conducted under different conditions. In moving worms, the RMD neurons oscillate differently. However, they showed significant bistable activity in electrophysiology. It may be due to the unique coding of three subclasses of RMD neurons (Lockery and Goodman, 2009), or it may be due to the multiple fixed points properties produced by the connection structure of RMD neurons. Our interpretation is that the existence of the dominant plateau potential in a network is the result of interaction between neurons (Lockery and Goodman, 2009). The multiple fixed points theory offers a possible explanation for the plateau potential in electrophysiology. Beside fixed point attractors, limit cycle is another kind of attractor, which may also occur in a neuronal net described by equation (2). Limit cycles are the attractors that may cause oscillation, which is worthy of further study in the future.

In recent years, control principles like the linear controllability theory has been used to study the function and neural activity of nematode connectome (Yan et al., 2017). However, the control principles used are difficult to correspond to biological implications, thus the resulting results lack biological interpretability compared with complex non-linear biological networks (Curto and Morrison, 2019; Jiang and Lai, 2019). In contrast, in our proposed structure-to-function research paradigm, we use a non-linear model to describe the neuronal activity of *C. elegans*. All parameters and functions in the model have specific biological meanings. The multiple fixed points features used in this study also have corresponding multistability phenomenon in animals, making the ultimate results much more biologically interpretable than the predicted results in Yan et al. (2017). This is what many previous studies lack (Curto and Morrison, 2019). In addition, previous studies adopted simplified network structure (Roberts et al., 2016) and substituted linear model for non-linear model (Liu et al., 2011; Yan et al., 2017) to avoid the computational complexity caused by non-linearity, whereas because the influence of the input and time-varying characteristics of the non-linear system is not needed for consideration in the study of fixed points, the difficulty of calculating the fixed points of the circuit is reduced. The functional deduction of ablation experiments shown in Table 3 has some similar results in Roberts et al., 2016. However, although there are significant differences between such neurons as AVB and PVC in the same locomotion function group, Roberts et al. (2016) cannot distinguish the function of these neurons, and would deduce that they have the same influence according to their method. By using a detailed structure that takes into account of every neuron in the circuit, we are able to analyze the unique role of each neuron and its connection.

As is shown in our proposed framework, bistable neurons can be obtained by calculating circuit dynamics in the structure-to-function research paradigm or by analyzing neuronal calcium activity data in the function-to-structure research paradigm. However, determining whether the dynamics of the circuit is caused by intrinsic properties or external factors, as well as its boundary or size, remains an obstacle. In a moving worm, the intrinsic dynamics of the circuit may not be the main cause of its neuronal activity. On the one hand, although most of the deduced results are still valid, some results of the first layer amphid interneuron circuit are not as satisfactory as those of the command circuit. The main reason is that the bistability of the command circuit is mainly caused by the interaction between neurons within this circuit, whereas the first-layer interneuron circuit receives complex input from sensory neurons and other interneurons outside this circuit, in addition to coupling with other bistable neurons. Although it is difficult to identify the non-bistable neurons involved in the neural activity of the first-layer interneuron circuit, bistable neurons tend to trigger a series of neural activities once they reach high potentials. On the other hand, because the locomotion of *C. elegans* is rather complicated, and forward–backward and turning behaviors are often coupled together (Gray et al., 2005), the possibility of other neurons participating in the structure-dominated functional process cannot be ruled out. Neurons such as AIBL/R also influence the reverse behavior besides turning (Gray et al., 2005). Other neurons such as RIML/R also play specific roles (Gray et al., 2005). These factors may all have an effect on our analysis results. Despite this, by separating the small circuits involved in pairwise behavior, such as forward and backward movements, fixed point theory helps to understand what role certain neurons play and how they promote certain functions. Fixed points indeed provide a critical link between the structure and functions of bistability related neuronal network, an essential step toward understanding the relationship between structure and function of small model organisms or their circuits. Therefore, in any case, once we obtain the fixed point characteristics needed for the analysis, we can obtain the satisfactory explanations regarding state switching and derivation of structural attributes from neuronal and circuit functions using our proposed framework. During this process, we use adjusted weight matrices in fixed-points calculation. By following the principles mentioned in Section “Method,” we can acquire the adjusted matrices utilized in fixed points analysis. However, the matrices are adjusted mainly manually, which means the results are to some extent arbitrary. We may get different matrices if we try the process again. In this paper, the main goal of the research is to provide a novel potential explanation for the emergence of multiple steady fixed points, instead of finding out the actual connection strength of the circuit. We do not use the connection weights to predict fixed points, but use fixed points to explain the phenomenon of multiples steady states. The adjusted matrices proves that the command circuit indeed is capable of producing multiple fixed points that are correspond to the locomotion behaviors. The followed analysis proves these fixed points can also provide satisfying function explanation. The goal of the study is fulfilled in this way. Of course, we had hoped to acquire the actual “strength” of connections. However, even after decades of study on *C. elegans*, there still lacks convincing conclusions on the signal transmission ability of synapses in *C. elegans*. Neither synaptic number or synaptic sizes can satisfy, although both of them are sometimes accepted as connection weights as presupposition conditions (Cook et al., 2019), it has never conclusively proved. We also tried to use learning algorithms for the weights of the adjacency matrices. But unfortunately, in moving worms, AVDL/R and PVCL/R are mostly actionless, making it difficult to study their connections from activities. We do hope that future studies on the synaptic strength can solve these problems. But in this study, the matrices have achieved their purpose of showing the ability for command circuit of producing multiple fixed points. Also, the exact excitatory and inhibitory properties of the synapses in *C. elegans* are still mostly uncertain. The excitatory and inhibitory properties we choose in this paper is based on experimental phenomenon (Chalfie et al., 1985) and previous research inference (Roberts et al., 2016). But there are also researches that provides different excitatory and inhibitory properties. Based on the study on the types of synaptic neurotransmitters and receptors, Rakowski and Karbowski (2017) and Fenyves et al. (2020) suggest a inhibitory dominant network among interneurons in *C. elegans*. Although we choose the excitatory and inhibitory properties as we present in this paper because some of the inhibitory synapses presented in these work (Rakowski and Karbowski, 2017; Fenyves et al., 2020) fail to provide reasonable explanation for known experimental phenomenon (Chalfie et al., 1985), it is still uncertain what the exact properties of these synapses are. We hope future studies will provide more detailed information on the neural network connection of *C. elegans*. Based on these, our fixed-point-based analytical framework mainly focus on the intrinsic dynamics of the circuit, which naturally provide more satisfactory structure deduction of circuits where internal signals dominate its activities.

Through the analysis of two research paradigms of the circuit of worms, it is shown that, in its free moving state, in addition to the stable fixed point corresponding to the transition between forward and backward states, we also find a third fixed point that is insignificant and short-lived compared with the forward and backward fixed point states. Using a computational analysis, all the solutions of these circuits obtained from Equation (3) contain a zero-vector solution (as shown in Table 1) corresponding to this fixed point that represents the long-term quiescent state of the worm, which emerges more often during the nematode’s sleeping state (Nichols et al., 2017). In sleeping worms, the fixed point corresponding to inactivity can persist for a long time (Nichols et al., 2017). Apparently, fixed points are dynamically changing according to different conditions, such as sleeping and awaking. Studies have shown that during sleep, certain substances are released to influence neurons (Lewis, 2014). As we have argued in the text, the third fixed point becomes a stable fixed point and has larger attraction domain in sleeping, which may be caused by the weaker information transmission efficiency of the chemical synapses during sleep, making the worm much more likely to remain inactive until the release of arousal stimulants terminates this process (Nichols et al., 2017).

Neuronal networks and their intrinsic multiple fixed points shape multistability, in which bistability is usually directly related to the function of the biological nervous system or its circuits (Kunert et al., 2015). Therefore, the fact that bistability is widespread in other animal neuronal networks besides *C. elegans* may imply that our analytical framework can be applied to other animal neural systems, including human. The main difference is that the neural networks of mammals are dominated by action potentials, while that of *C. elegans* is dominated by gradient potentials. If the fixed points of a mammalian neuronal network can be calculated, similar analytical process can follow our proposed framework to gain the deeper insight into the relationship between function and structure. On the whole, based on the fixed point theory, our proposed analytical framework opens up a new perspective for the study of structure-function relationship, especially through the tools of non-linear dynamics and neural data analysis, to reveal the attractor characteristic inherent in neural circuits in different ways, promoting the interpretability of neuronal function and the reliability of underlying structure deduction. Moreover, since we focus on the multistability phenomenon caused by the intrinsic interaction among neurons, this framework is expected to be applied to interpret the neural activity patterns in spontaneous activities or resting states. Apart from fixed points, attractors such as limit cycles are also of promising research value for understanding the neural dynamics of *C. elegans* and other animals, which warrants future studies.

## Method

### Model Parameter Setting

In order to make the model of the command circuit we study emerge the expected non-linear dynamic characteristics of the fixed point, we need to consider the parameter setting of the model in computation.

For chemical synapses in Equation (2), three parameters are involved. First, since we study neurons of similar types such as command neurons, we assume that the time constant is the same without affecting the results of the study. Then the time constant τ in Equation (3) is neutralized, having no influence on the value of fixed points mathematically. For convenience, we set τ=1. Second, the other two are related to the sigmoidal function. In order to make the Equation (2) better describe the chemical synapses potential of *C. elegans*, we need to set proper threshold θ and high slope *k* of the sigmoidal function. So, we have θ=0.5 and *k* = 20 in our fixed point calculation.

### Adjustment of the Adjacency Matrices of Electrical and Chemical Synapses

Original connectome data can be acquired from Wormwiring^1^ (Cook et al., 2019). In chemical and gap junctional neuronal networks of the command circuit, excitatory or inhibitory synapses and their strength are important network properties for the study of their non-linear dynamics. The synaptic weight simply measured by the number (or plus size) of chemical or gap junction synapses (Cook et al., 2019) cannot better reflect the connection strength between each neuron pair, let alone the excitability or inhibitory properties between them. For an example, there are much more, nearly twice more electrical synapses between AVA and PVC than those between AVAL and AVAR. But obviously, AVA and PVC do not show synchronization activities at all while AVAL and AVAR are exactly synchronous. Apparently, the electrical synaptic strength between AVA and PVC are rather low despite the large number of electrical synapses between them. Obviously, it is difficult to obtain the actual chemical and gap junctional strengths of the command circuit. Therefore, in order to make this simulated circuit emerge the desired non-linear dynamic characteristics, such as multiple fixed points, we use its adjacency matrix (Cook et al., 2019) as a reference to obtain the connection weight matrix of the command circuit (see Supplementary Information II).

To do this, in addition to considering the number of synapses (for the sake of generality, we ignore the size of the synapse here), we follow the following rules. (1) The chemical connections within the same section are excitatory and have positive weights, while the chemical synapses between two sections are inhibitory and have negative weights. In order for neurons to activate or inhibit other neurons through chemical synapses, we set the maximum and minimum values of chemical synaptic strength to be 1 and −1, respectively. (2) The weight of gap junction between a pair of neurons in the same class is rather large, up to 1 or 2 mostly, to ensure the synchronization activities of the neuron pairs. (3) The weight of gap junctions within the same section is more than that between different sections to ensure opposite activities. (4) The more the number of chemical and gap junctional synapses, the greater the connection weight tends to be. For the electrical synapses in Equation (2), larger electrical synaptic strength between pairs of neurons can ensure their synchronous activities. If neurons do not synchronize with each other, their electrical synaptic strength should be reduced. In addition, some connections with only one or two synapses have little impact on the circuit behavior, so we set the weights of these connections to around 0.1 to maintain their existence and influence.

For the command circuit, the original adjacency matrices of chemical and electrical synapses are presented in Supplementary Tables 1, 2, respectively. After adjustment, the adjacency matrices of chemical and electrical synapses are presented in Supplementary Tables 7, 8, respectively. Although adjustment is to some extent variable, it is enough to serve the purpose of providing a novel potential explanation for the existence of multiple stable neural states and how they switch.

### Determination of Fixed Points

#### Computing From Non-linear Neuronal Network

The adjusted adjacency matrices *W* and *G* obtained by using the method above that can be utilized to compute fixed points. Put them into Equation (3), and set the parameters τ=1, θ=0.5 and *k* = 20, the solutions to Equation (3) are then the fixed points of the circuit calculated.

### Estimation of Fixed Points From Calcium Activity

Based on the function-to-structure research paradigm, we first looked to see if there was any stability in the calcium activity data. A neuron is considered stable if its calcium activity remains relatively stable within a certain range over a considerable period. From all the stable performances of all neurons, we acquire pattern combination activities, some of which are always active and some of which are always not. Therefore, these pattern behaviors can be determined as multiple fixed points.

### Approximation of Calcium Activity of Neurons by Membrane Potential

Studies in Kato et al. (2015) have shown that the calcium activity measured by the electrophysiological preparation are highly consistent with the membrane voltage, which allows us to use calcium activity to represent the activity of membrane potential.

### *C. elegans* Dataset

The neuronal network connectome of C. *elegans* is collected from the website Wormwiring^2^.

### Experimental Verification Methods

#### Worm Maintenance, Calcium Imaging, and Laser Ablation

All worms were raised at 20°C on NGM plates seeded with OP50 *Escherichia coli* as previously described (Brenner, 1974).

Whole brain calcium imaging was conducted as described in Yemini et al. (2020). Animals (strain OH16230) were immobilized and mounted on 2% agarose pad after 45 min treatment of 1 mM tetramisole. Whole-brain calcium activity was imaged using an inverted spinning disk confocal with 40× water immersion objective. NeuroPAL imaging was conducted with 4 different laser lines (405, 488, 560, and 647 nm).

A 10-min recording of the panneuronal, nuclear GCaMP6s activity was captured at approximately 1–2 volume per second using a laser line of 488 nm.

Laser ablation was performed on L1–L2 animals using MicroPoint Laser system (Andor – Oxford Instruments). Animals after laser ablation were allowed to grow to D1 adult for behavioral testing. Transgenic line Pnmr-1::gfp was used to ablate AVA, AVE, AVD, and PVC neurons, and transgenic line Pacr-15(a)::gfp was used to ablate AVB neurons.

## Data Availability Statement

The datasets presented in this study can be found in online repositories. The names of the repository/repositories and accession number(s) can be found in the article/Supplementary Material.

## Author Contributions

JL and TF conceived the project and design the research process. JL, ZL, and TF designed the biological experiments, which were mostly performed by WL. HH checked the feasibility of the research. JL processed theoretical reasoning, constructed the analytical framework, finished the computing work, and analyzed the computing date. WL and KX analyzed the biological experimental date. JL and KX plotted the pictures. JL, YY, and PZ did the research work on state machines. WL wrote a portion of the method. HH, ZL, and TF jointly supervised this work. All authors helped to wrote the manuscript.

## Conflict of Interest

The authors declare that the research was conducted in the absence of any commercial or financial relationships that could be construed as a potential conflict of interest.

## Publisher’s Note

All claims expressed in this article are solely those of the authors and do not necessarily represent those of their affiliated organizations, or those of the publisher, the editors and the reviewers. Any product that may be evaluated in this article, or claim that may be made by its manufacturer, is not guaranteed or endorsed by the publisher.

## Footnotes

1
https://wormwiring.org/

2
https://wormwiring.org/pages/adjacency.html
