# Supplementary material for: Fixed Point Attractor Theory Bridges Structure and Function in C. elegans Neuronal Network
Source: Front Neurosci. 2022 Apr 25;16:808824. doi: 10.3389/fnins.2022.808824 (PMC9085386; doi:10.3389/fnins.2022.808824)
Supplement: Supplementary file 1 [file Table_1.pdf]

Fixed Point Attractor Theory Bridges Structure and Function in C. *elegans* Neuronal Network

Supplementary Information

I. C. *elegans* connectome

Our study works mainly focus on two circuits: command circuit, the first layer amphid interneurons. The connectome date of C. *elegans* is acquired from the website Wormwiring and corresponding papers[1-3].

A. Nematode command circuit

In C. *elegans*, there are distinct command neurons, for example, HSNL/R neurons initiating egg laying[4], RIML/R neurons which are generally considered to be the pre-command interneurons and have strong influence on forward/reverse locomotion[5]. It is generally believed that the nematode command circuit is composed of 10 neurons, namely AVBL/R, PVCL/R, AVAL/R, AVDL/R and AVEL/R neurons, which play an important role in the forward/reverse locomotion behavior of nematode[6]. The structure of command circuit used in our study are shown in Fig.3(a) in the body of the paper. According to the data collected from Wormwiring[3], the adjacency matrices of the chemical synapses and gap junctions of the command circuit is shown in supplementary table 1and supplementary table 2, respectively.

Supplementary Table 1. The adjacency matrix of the chemical synapses of the command circuit.

	AVBL	AVBR	PVCL	PVCR	AVAL	AVAR	AVDL	AVDR	AVEL	AVER
AVBL	0	3	0	1	8	12	0	2	1	1
AVBR	1	0	0	0	6	7	3	0	0	2
PVCL	6	14	1	3	5	5	6	4	2	1
PVCR	7	9	5	0	7	5	5	4	1	1
AVAL	1	0	12	9	0	9	2	2	1	1
AVAR	3	2	9	6	7	0	2	5	2	2
AVDL	0	0	1	0	23	24	0	3	3	0
AVDR	1	0	0	2	25	36	3	0	0	2
AVEL	0	0	1	1	11	7	3	0	0	0
AVER	0	0	0	0	8	19	2	2	1	0

Supplementary Table 2. The adjacency matrix of the gap junctions of the command circuit.

	AVBL	AVBR	PVCL	PVCR	AVAL	AVAR	AVDL	AVDR	AVEL	AVER
AVBL	0	8	0	0	0	1	0	0	0	0
AVBR	8	0	0	0	0	0	0	0	0	0
PVCL	0	0	0	27	3	7	0	0	0	0
PVCR	0	0	27	0	4	11	0	1	0	0
AVAL	0	0	3	4	0	8	3	5	2	0
AVAR	1	0	7	11	8	0	1	8	0	1

AVDL	0	0	0	0	3	1	0	4	0	0
AVDR	0	0	0	1	5	8	4	0	0	0
AVEL	0	0	0	0	2	0	0	0	0	1
AVER	0	0	0	0	0	1	0	0	1	0

B. First layer amphid interneurons

The first layer amphid interneuron circuit in C. *elegans* consists of AIAL/R, AIYL/R, AIBL/R and AIZL/R paired neurons. They receive and process information from the amphid sensory neurons[7,8].

The numbers in the matrices are the number of synapses discovered.

The adjacency matrix of chemical synapses of first layer amphid interneurons is:

Supplementary Table 3. The adjacency matrix of the chemical synapses of the first layer amphid interneurons.

	AIAL	AIAR	AIYL	AIYR	AIBL	AIBR	AIZL	AIZR
AIAL	0	0	0	0	0	0	0	0
AIAR	0	1	0	0	0	19	0	1
AIYL	0	0	0	0	3	0	21	0
AIYR	0	0	2	0	0	0	0	17
AIBL	0	0	0	0	1	0	0	3
AIBR	0	1	0	1	0	0	0	0
AIZL	0	0	0	0	6	24	0	0
AIZR	0	5	0	0	15	3	0	0

Supplementary Table 4. The adjacency matrix of the gap junctions of the first layer amphid interneurons.

	AIAL	AIAR	AIYL	AIYR	AIBL	AIBR	AIZL	AIZR
AIAL	0	0	0	0	0	0	0	0
AIAR	0	1	0	0	0	0	0	0
AIYL	0	0	0	1	0	0	0	0
AIYR	0	0	1	0	0	0	0	0
AIBL	0	0	0	0	0	0	0	2
AIBR	0	0	0	0	0	0	2	0
AIZL	0	0	0	0	0	2	0	2
AIZR	0	0	0	0	2	0	2	0

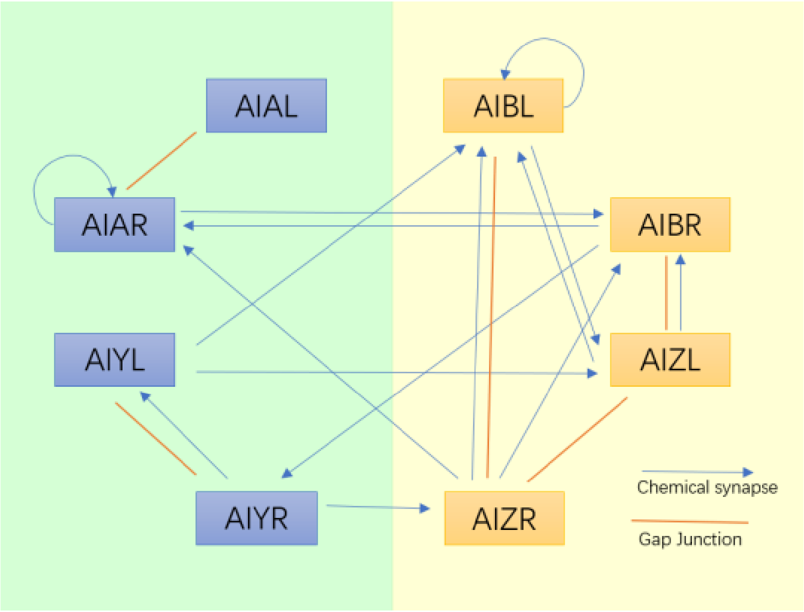

Supplementary figure 1 The connectome of first layer amphid interneurons.

C. RMD class

These RMD neurons consist of six neurons, namely RMDDL, RMDDR, RMDL, RMDR, RMDVL and RMDVR. The adjacency matrices of RMD neurons are shown in Supplementary Table 5 and Supplementary Table 6.

Supplementary Table 5. The adjacency matrix of the chemical synapses of the RMD neurons.

	RMDDL	RMDDR	RMDL	RMDR	RMDVL	RMDVR
RMDDL	0	0	0	0	1	9
RMDDR	1	0	0	0	9	1
RMDL	0	4	0	5	2	2
RMDR	3	0	5	0	4	0
RMDVL	0	10	0	1	0	1
RMDVR	20	0	0	0	0	2

Supplementary Table 6. The adjacency matrix of the gap junctions of the RMD neurons.

	RMDDL	RMDDR	RMDL	RMDR	RMDVL	RMDVR
RMDDL	0	0	1	0	1	0
RMDDR	0	0	0	0	0	2
RMDL	1	0	0	0	1	0
RMDR	0	0	0	0	0	1
RMDVL	1	0	1	0	0	0
RMDVR	0	2	0	1	0	0

II. Adjusting and discussing the adjacency matrix of the command circuit

The change in the weight matrix of the command circuit is one of the important factors to make this command circuit with a given connection structure emerge the desired specific dynamic characteristics. Since the neurons in command circuit are rather densely connected, the weight of one edge may have a great influence on the dynamics of the circuit. According to previous studies, we have the number of synapses between neurons, and even weights considering both number and size of synapses[3]. Unfortunately, the magnitude of weights still ranges from one to dozens. If we use these data in the module, we will have extraordinarily high signal in some edges. Studies have shown that the number of synapses cannot represent the strength of neuron connections. Therefore, all of the connections need to be limit to certain ranges.

However, the adjustment process is complicated. Neither linear scaling nor nonlinear scaling can obtain satisfactory results for adjacency matrix. There is no better way to optimize these weight matrix parameters at present. Here we start from the adjacency matrix of the command circuit and adjust the connection weight matrix of the command circuit in a trial and error way to gradually make the fixed points appear in the command circuit.

The main rules of the adjustment are shown in Method. At each adjustment, we calculate the fixed points of this circuit with equation (3) shown in main text. This manual process of trial and error may vary from person to person, but as long as the rules are followed and the three desired points showing the correct activation and inactivation states are obtained, the interpretation results remain still satisfactory, without affecting the subsequent structure-function analysis and neuronal function analysis.

The function of the neurons in terms of their contribution to fixed points and attraction are still valid. We have the adjusted chemical adjacency matrix as Supplementary Table 7.

Supplementary Table 7. The adjacency matrix of the chemical synapses of the command circuit after adjustment.

	AVBL	AVBR	PVCL	PVCR	AVAL	AVAR	AVDL	AVDR	AVEL	AVER
AVBL	0	1	0	0.1	-1	-1	0	-0.2	-0.1	-0.1
AVBR	1	0	0	0	-1	-1	-0.2	0	0	-0.1
PVCL	2	1	0.1	0.5	-1	-1	-1	-1	-1	-1
PVCR	2	2	0.5	0	-1	-1	-1	-1	-1	-1
AVAL	-0.5	0	-1	-1	0	1	0.05	0.05	0.1	0.1
AVAR	-1	-1	-1	-1	1	0	0.05	0.05	0.1	0.1
AVDL	0	0	-0.1	0	1	1	0	0.1	0.1	0
AVDR	-0.1	0	0	-0.1	1	1	0.1	0	0	0.1
AVEL	0	0	-0.1	-0.1	1	1	0.05	0	0	0
AVER	0	0	0	0	1	1	0.05	0.05	0.1	0

And the gap junction adjacency matrix as

Supplementary Table 8. The adjacency matrix of the gap junctions of the command circuit after adjustment.

	AVBL	AVBR	PVCL	PVCR	AVAL	AVAR	AVDL	AVDR	AVEL	AVER
AVBL	0	2	0	0	0	0.1	0	0	0	0
AVBR	2	0	0	0	0	0	0	0	0	0
PVCL	0	0	0	5	0.3	0.3	0	0	0	0
PVCR	0	0	5	0	0.3	0.3	0	0.1	0	0
AVAL	0	0	0.3	0.3	0	2	0.1	0.1	1	0
AVAR	0.1	0	0.3	0.3	2	0	0.1	0.1	0	1
AVDL	0	0	0	0	0.1	0.1	0	1	0	0
AVDR	0	0	0	0.1	0.1	0.1	1	0	0	0
AVEL	0	0	0	0	1	0	0	0	0	1
AVER	0	0	0	0	0	1	0	0	1	0

III. C. *elegans* nonlinear neuronal network model

In this paper, the neuronal circuit we obtained from the nematode's connectome is a neuronal network, such as the command circuit or the first layer amphid interneurons (AIY-AIZ-AIB-AIA), according to its specific function. For such a neural network, we use the nonlinear differential method [gradient potential] as shown in Equation (1) in the text to describe the dynamics of each neuron, and the network thus constructed has the characteristics of nonlinear dynamics. Here, in the text, Equation (2) is deduced as follows.

Assuming a neurons network consisting of N neurons following the dynamic description of equation (1), which, for the simplicity of argument, have the same time constants τ and releasing parameters θ, the neuronal network can be described as follows:

$$\tau\begin{bmatrix}\dot{x_1}\\ \dot{x_2}\\ \vdots\\ \dot{x_n}\end{bmatrix}=-\begin{bmatrix}x_1\\ x_2\\ \vdots\\ x_n\end{bmatrix}+\begin{bmatrix}\sum_{j=1}^{N}w_{j1}\sigma(x_j-\theta)\\ \sum_{j=1}^{N}w_{j2}\sigma(x_j-\theta)\\ \vdots\\ \sum_{j=1}^{N}w_{jn}\sigma(x_j-\theta)\end{bmatrix}+\begin{bmatrix}\sum_{k=1}^{N}g_{k1}(x_k-x_1)\\ \sum_{k=1}^{N}g_{k2}(x_k-x_2)\\ \vdots\\ \sum_{k=1}^{N}g_{kn}(x_k-x_n)\end{bmatrix}+Bu \qquad \text{(S1)}$$

where Bu represents the external input.

The chemical synapses input can be rewrite as:

$$\begin{bmatrix}\sum_{j=1}^{N}w_{j1}\sigma(x_j-\theta)\\ \sum_{j=1}^{N}w_{j2}\sigma(x_j-\theta)\\ \vdots\\ \sum_{j=1}^{N}w_{jn}\sigma(x_j-\theta)\end{bmatrix}=\begin{bmatrix}w_{11}&w_{21}&\cdots&w_{n1}\\ w_{12}&w_{22}&\cdots&w_{n2}\\ \vdots&\vdots&\ddots&\vdots\\ w_{1n}&w_{2n}&\cdots&w_{nn}\end{bmatrix}\begin{bmatrix}\sigma(x_1-\theta)\\ \sigma(x_2-\theta)\\ \vdots\\ \sigma(x_n-\theta)\end{bmatrix},$$ and the gap junction input as:

$$\begin{bmatrix}\sum_{k=1}^{N}g_{k1}(x_k-x_1)\\ \sum_{k=1}^{N}g_{k2}(x_k-x_2)\\ \vdots\\ \sum_{k=1}^{N}g_{kn}(x_k-x_n)\end{bmatrix}=\begin{bmatrix}\sum_{k=1}^{N}g_{k1}x_k\\ \sum_{k=1}^{N}g_{k2}x_k\\ \vdots\\ \sum_{k=1}^{N}g_{kn}x_k\end{bmatrix}-\begin{bmatrix}\sum_{k=1}^{N}g_{k1}x_1\\ \sum_{k=1}^{N}g_{k2}x_2\\ \vdots\\ \sum_{k=1}^{N}g_{kn}x_n\end{bmatrix}=\begin{bmatrix}g_{11}&g_{21}&\cdots&g_{n1}\\ g_{12}&g_{22}&\cdots&g_{n2}\\ \vdots&\vdots&\ddots&\vdots\\ g_{1n}&g_{2n}&\cdots&g_{nn}\end{bmatrix}\begin{bmatrix}x_1\\ x_2\\ \vdots\\ x_n\end{bmatrix}-$$

$$\begin{bmatrix}\sum_{k=1}^{N}g_{k1}&0&\cdots&0\\ 0&\sum_{k=1}^{N}g_{k2}&\cdots&0\\ \vdots&\vdots&\ddots&\vdots\\ 0&0&\cdots&\sum_{k=1}^{N}g_{kn}\end{bmatrix}\begin{bmatrix}x_1\\ x_2\\ \vdots\\ x_n\end{bmatrix}.$$

Combining these three equations, we have C. *elegans* nonlinear neuronal network model as

follows:

$$\tau\dot{x} = (G - L - E_{n\times n})x + W\sigma(x-\theta) + Bu$$

or:

$$\dot{x} = \frac{1}{\tau}(G - L - E_{n\times n})x + \frac{1}{\tau}W\sigma(x-\theta) + \frac{1}{\tau}Bu \qquad \text{(S2)}$$

where $x = [x_1, x_2, \dots, x_n]^T$, $\sigma(x-\theta) = [\sigma(x_1-\theta), \sigma(x_2-\theta), \dots, \sigma(x_n-\theta)]^T$, $W = \begin{bmatrix} w_{11} & w_{21} & \cdots & w_{n1} \\ w_{12} & w_{22} & \cdots & w_{n2} \\ \vdots & \vdots & \ddots & \vdots \\ w_{1n} & w_{2n} & \cdots & w_{nn} \end{bmatrix}$, $G = \begin{bmatrix} g_{11} & g_{21} & \cdots & g_{n1} \\ g_{12} & g_{22} & \cdots & g_{n2} \\ \vdots & \vdots & \ddots & \vdots \\ g_{1n} & g_{2n} & \cdots & g_{nn} \end{bmatrix}$, and $L = diag(\sum_{k=1}^{n} g_{k1}, \sum_{k=1}^{n} g_{k2}, \dots, \sum_{k=1}^{n} g_{kn})$.

Equation (2) concisely and clearly describes the nonlinear dynamic characteristics of C. *elegans* neuronal networks.

IV. Proof of sufficiency theorem for motifs with multiple fixed points

Since a multiple fixed points motif can enable other neurons connected to it to obtain similar properties, we only need to study the basic situation that all neurons in the motif are part of the feedback loop. The rate of change of states at fixed points is zero, meaning $\dot{x} = 0$. According to Equation (3) in the text, we have:

$$(E + L - G)x = W\sigma(x-\theta) \qquad \text{(S3)}$$

As is mentioned earlier, the fixed point of resting potential exists regardless of the connections. The fixed point around the threshold of sigmoidal function is an unstable fixed point, making it very difficult to achieve. The fixed points we focus on are the ones caused by the saturation distribution of sigmoidal function. Since the output range of sigmoidal function is $[0, 1]$, at the fixed point we studied, we have:

$$\sigma(x_i - \theta) = 1 \qquad \text{(S4)}$$

This means that the potential of each node is above the sigmoidal threshold:

$$x_i > \theta, \qquad i = 1,2, \dots, n \qquad \text{(S5)}$$

In Equation (S3), both L and G are nonnegative diagonal matrices. $E_{n\times n}$ is a unit matrix. Therefore, $(E_{n\times n} + L - G)$ is a symmetric matrix, whose diagonal elements are greater than or equal to 1, off-diagonal elements are less than or equal to 0. Obviously, matrix $(E_{n\times n} + L - G)$ is nonsingular and invertible. Therefore, Equation (S3) can be rewritten as:

$$x = (E_{n\times n} + L - G)^{-1} W\sigma(x-\theta) \qquad \text{(S6)}$$

Because the fixed point we study is the near (3.1.2), we have:

$$x = (E_{n\times n} + L - G)^{-1} W \begin{bmatrix} 1 \\ 1 \\ \vdots \\ 1 \end{bmatrix} \qquad \text{(S7)}$$

If equation (S7) satisfies Equation (S5), X is a nonzero vector, which means there is a fixed point with a nonzero vector. What we need to prove is how to meet this condition.

Since matrix $(E_{n\times n}+L-G)$ is symmetrical, G is a non-negative symmetrical matrix and $L=diag(\sum_{k=1}^{n}g_{k1},\sum_{k=1}^{n}g_{k2},\dots,\sum_{k=1}^{n}g_{kn})$, it is easy to derive that all the elements in $(E_{n\times n}+L-G)^{-1}$ are nonnegative and the diagonal elements in L are definite positive. Here we discuss three cases.

Case 1: Feedback loop constructed by chemical connections.

If the feedback loop of n neurons has a complete chemical series connection, the chemical connection matrix can be written as

$$W=\begin{bmatrix} 0 & w_{21} & * & \cdots & * \\ * & 0 & w_{32} & \cdots & * \\ * & * & 0 & \ddots & \vdots \\ \vdots & \vdots & \ddots & 0 & w_{n,n-1} \\ w_{1n} & * & \cdots & * & 0 \end{bmatrix}_{n\times n}$$

where w_{ij} are the weights of the excitatory chemical synapses that construct the positive feedback loop and they are all positive. The diagonal elements are zero, and other elements written as $*$ are unknown.

The general rank of matrix w is n. Under the conditions proposed in the theorem, that is, given $w_{ij}>0$, w_{ij} here can be arbitrarily adjusted, state vector x obtained from Equation (S7) can easily satisfy Equation (S5).

Case 2: Feedback loops constructed by combining chemical synapses with electrical synapses, but not by existing chemical synapses alone.

In this case, the chemical connection matrix can be written as

$$W=\begin{bmatrix} 0 & w_{21} & * & \cdots & * \\ * & 0 & w_{32} & \cdots & * \\ * & * & 0 & \ddots & \vdots \\ \vdots & \vdots & \ddots & 0 & w_{n,n-1} \\ w_{1n} & * & \cdots & * & 0 \end{bmatrix}_{n\times n}$$

where the weights w_{ij} are an adjustable non-negative element, and cannot all be zero.

Presumably the $w_{pq}=0$, meaning that there is no chemical connection from neuron q to neuron p. According to the condition in case 2, there is a gap junction between these two neurons. Therefore, the corresponding elements in the gap connection matrices $G(p,q)$ and $G(q,p)$ are positive. Judging from the characteristic of adjoint matrix, the corresponding $(E_{n\times n}+L-G)^{-1}(p,q)$ and $(E_{n\times n}+L-G)^{-1}(q,p)$ must be positive.

Since the weights w_{ij} are positive except $w_{pq}=0$, the pth element in the $n\times 1$ column

vector $W\begin{bmatrix}1\\1\\\vdots\\1\end{bmatrix}$ is unknown, but the other elements are controllable by adjusting w_{ij}. And because $(E_{n\times n}+L-G)^{-1}$ is non-negative, $(E_{n\times n}+L-G)^{-1}(p,q)$ and $(E_{n\times n}+L-G)^{-1}(q,p)$ are both positive, all elements in $x=(E_{n\times n}+L-G)^{-1}W\begin{bmatrix}1\\1\\\vdots\\1\end{bmatrix}$ can be adjusted to be positive, satisfying condition described by Equation (S5).

Case 3: Feedback loop purely constructed by gap junctions.

When gap junctions form a complete feedback loop, we have the gap connection matrix

$$G=\begin{bmatrix} 0 & g_{21} & & * & \cdots & g_{n1} \\ g_{12} & 0 & & g_{32} & \cdots & * \\ * & g_{23} & 0 & \ddots & & \vdots \\ \vdots & \vdots & \ddots & 0 & & g_{n,n-1} \\ g_{1n} & * & \cdots & g_{n-1,n} & & 0 \end{bmatrix}_{n\times n}$$

Note that G is a positive symmetric matrix, $g_{ij}=g_{ji}$. Matrix G is then general full rank. Therefore, the general rank of $(E_{n\times n}+L-G)$ is n. Since a complete gap junction connection is established, if there are two neurons connecting each other through chemical synapses, this would qualify the condition in Case 2. In case 3, chemical connection matrix $W=0$. Therefore, $(E_{n\times n}+L-G)^{-1}W=0$. Then Equation (S6) is a zero vector and cannot satisfy Equation (S5).

The theorem is proved.

To sum up, excitatory chemical synapses, which dominate the positive feedback loop, plays a crucial role in the existence of multiple fixed points in the neuronal networks. Gap junctions contribute to neuronal synchronization but do not help maintain multiple fixed points. Multiple fixed point motifs connect other organizations and each other through chemical synapses and gap junctions, making more complex performance.

V Fixed points based state machine

A state machine consists of two elements: states and conditions. We use multiple fixed points as states and the attraction domains as switching conditions to construct a state machine, as shown in Fig.2 in the body of the paper. With this mechanism, a neuronal network with n fixed points will be able to switch freely among n states according to the attraction domains. This helps to understand how C. *elegans* respond to external environment, maintain behaviors and switch among them. Fixed points are the default states of the neuronal network. Each of these states may be related to a specific function or behavior. When the system receives external input or internal noise, its state will drift from its previous fixed point. When the disturbance terminates, it will

drift back to this fixed point if the state is at still within the previous attraction domain. If it reaches another attraction domain, the state will switch to the corresponding fixed point instead. This mechanism allows the system to reach preset states and switch among them accordingly. The behavior of a C. *elegans* is continuous and the output signals are varying. In the case of unstable input, it is important to maintain internal stable activity of the neurons and to be able to switch to another desired state when needed.

The state drifts may be caused by external input and internal noise. Both of them all exist in C. *elegans* and play important roles in maintaining the normal function of neuronal networks. We suggest that state drift caused by internal noise may be the reason for spontaneous random behaviors of C. *elegans*. The state drift caused by changes in external inputs is the key to responding to significant environmental changes. Both of these situations can be observed in the neuronal activity of C. *elegans*. According to this theory, we can understand the function of neurons in terms of both states (multiple fixed points) and conditions (attraction domains). We found that some neurons are essential for the existence of multiple fixed points, and others may contribute to the formation of the attraction domains of fixed points.

Forward-reverse witching of C. *elegans* under stimulation is obviously the result of state drift caused by external input. While random switches occur when no significant stimulation is imposed on the worm. Thus, it might be argued that this could be the result of internal noise. Then we have the command circuit dynamic (2) rewritten as

$$\dot{x} = \frac{1}{\tau}(G - L - E_{n\times n})x + \frac{1}{\tau}W\sigma(x-\theta) + \frac{1}{\tau}Bu + Noise$$

Noise within the system allows the state to change from the fixed point. If it locates in the domain of attraction of another fixed point, switch happens. The activities of the circuit are complicated, constructing a high dimensional space. It is mathematically difficult to calculate the domain of attraction due to the complexity of the nonlinear system. In practice, we only need to calculate the final state under a certain initial state. If the final state is one of the fixed points, then the initial state is within its corresponding attraction domain.

VI. Prediction process of the structural properties of the first layer interneurons

When predicting the structural properties of the first layer amphid interneurons of C. *elegans*, we can qualitatively obtain some specific functions and some specific dynamic properties (see Supplementary figure 2 (a)) of the neurons that make up this circuit by comparing and analyzing the observed data of calcium activity of each neuron in the circuit. First, AIAL/R and AIZL/R have little activities, while AIAL/R and AIYL/R have opposite activities. Second, in terms of neuron functions, AIAL/R, AIYL/R inhibits turns, while AIBL/R and AIZL/R promote turns[10]. Third, in terms of dynamics, AIBL/R and AIYL/R showed obvious bistability. From the above qualitative description, we can reasonably infer that AIAL/R and AIVL/R have the dynamic characteristics of multiple fixed points. According to the influence of these neurons on behaviors,

they were divided into two opposed groups, namely Group AIAL/R, AIYL/R, and group AIBL/R, AIZL/R. According to the activity patterns of the neurons, in addition to the resting potential fixed point, there are also two, thus constructing the state machine of this circuit, shown in Supplementary figure 2 (b).

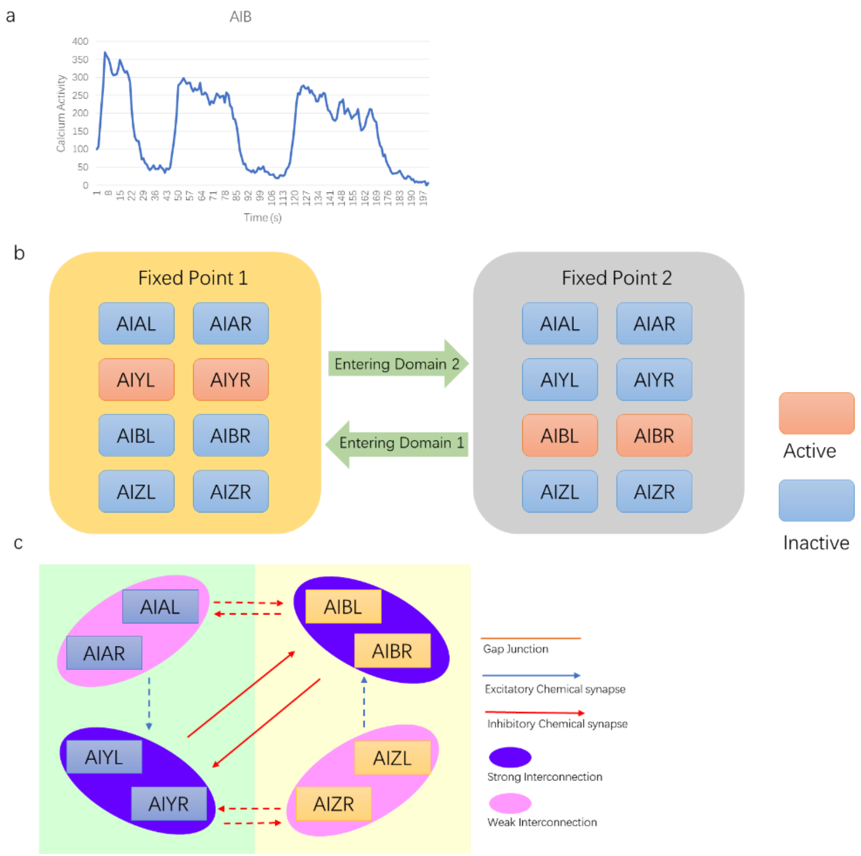

Supplementary figure 2 First layer amphid interneurons include AIAL/R, AIBL/R, AIYL/R and AIZL/R. (a), Neuronal activities. (b), Fixed points based state machine. (c), Deducing possible connectome (connections).

The state switching of this circuit mainly occurs between these two fixed points (excluding the fixed point where all neurons stay inactive). As neurons with bistability play vital roles in maintaining the state, we further extrapolate the roles of these neurons: AIYL/R and AIBL/R give rise to the associated attractors, and AIAL/R and AIZL/R enlarges the attraction domains. According to our proposed theorem, the strong excitatory chemical feedback loop promotes the existence of multiple fixed points. Therefore, we can deduce that AIYL/R and AIBL/R tend to have strong interconnections, while AIAL/R and AIZL/R tend to have relatively weak interconnections. From the fact that these two groups of neurons (AIYL/R and AIBL/R) have opposite bistable states, it is possible that they inhibit each other. Therefore, it is most likely that AIBL/R and AIYL/R are interconnected with strong inhibitory chemical interactions.

AIAL/R and AIZL/R enlarge the attraction domains of the corresponding fixed points. There are probably three ways to achieve this. They can either activate the same group of neurons that

maintain a state, or inhibits those that maintain an opposite state, or both. The neurons that are bistable are unlikely to activate those that are not, but may inhibit them. Therefore, the deducing process from functional activities to structural properties is shown in Supplementary figure 2. (c).

Based on the fixed point attractor, we establish linked relationship among structure, dynamic and function, thus we derive these possible results in Table 3 of the body of the paper.

VII. Experimental verification

A. Three locomotory states in sleeping animals

In addition to the observation of moving worm as shown in Fig. 4 in the body of the paper, we also conduct the following locomotion observation of sleeping worms. The resting state is significantly more obvious than that of moving worms, while the reverse behavior is quite rare. The three states of forward, reverse and pause behaviors are common in C. *elegans*, but their distributions are different in moving and sleeping individuals.

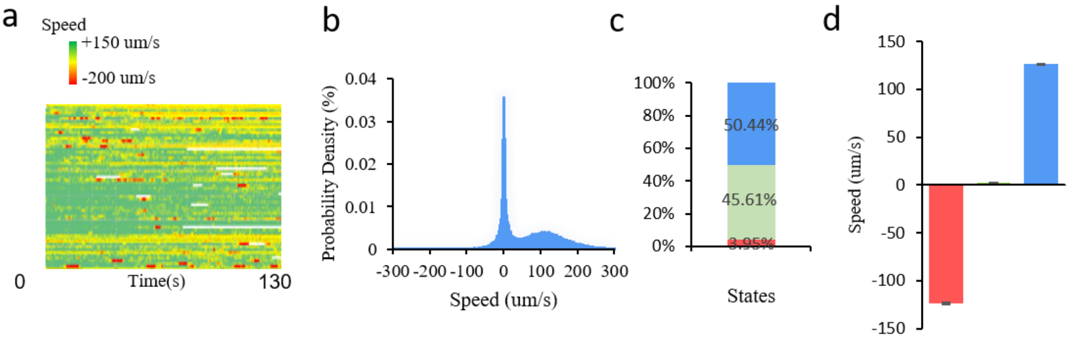

Supplementary figure 3. Three locomotory states in sleeping animals: forward, reverse and pause**.**

(a) Speed heat map of a group of worms. Each row represents a speed trace, and green and red represent forward and backward speeds, respectively. (b) Probability density of speed distribution. Bin size = 1. (c) The percentage of three locomotory states. Forward movement accounts for 50.44% of locomotory time, resting state accounts for 45.61% of locomotory time and backward movement only accounts for 3.95%. (d) Average velocity of three states. Control group T-test, $p < .001$. Differences between backward and resting states, T-test, $p < .001$. Differences between forward and resting states, T-test, $p < .001$. Differences between Backward and forward states, T-test, $p < .001$.

B. Multiple states in C. *elegans* neural activity with resting state.

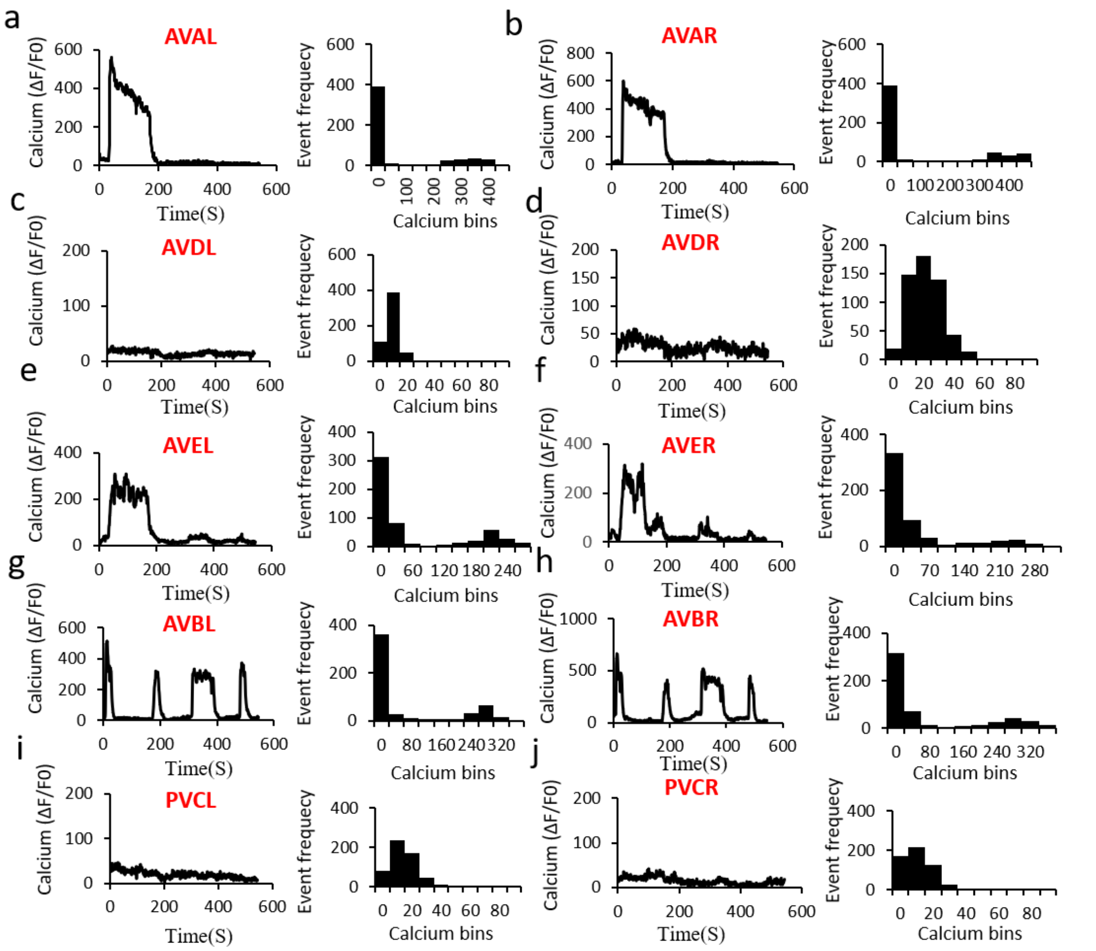

Supplementary figure 4**.** The neural activity of AVAL (a), AVAR (b), AVDL(c), AVDR (d), AVEL (e), AVER (f), AVBL (g), AVBR (h), PVCL (i) and PVCR (j). Left panel shows change in calcium activity over time. Right panel shows the distribution of changes in calcium activity. Bin size = 50 (a), 50 (b), 10 (c), 10 (d),30 (e), 35 (f), 40 (g), 40 (h), 10 (i), 10 (j).

Compared with the data of moving worms, the pausing state, where command neurons stay inactive, is more obvious in resting worms[11]. Forward-reverse-pause switch is more complete when moving and resting states are both taken into account.

C. Multiple states in C. *elegans* neural activity when moving.

The neural activity of C. *elegans* during another period of time different from Fig.5 in the body of the paper.

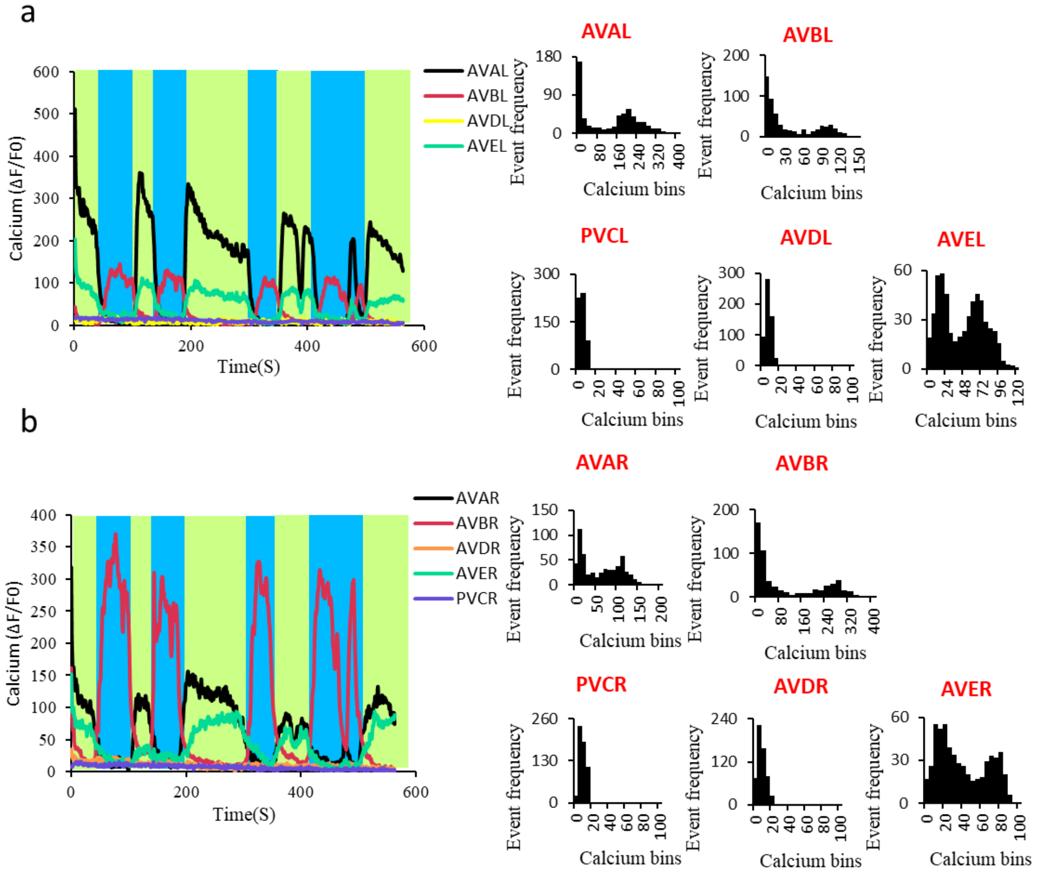

Supplementary figure 5. Two states in C. *elegans* neural activity. (a) The neural activity of AVAL, AVBL, AVDL, AVEL, and PVCL. The graph at the left of the panel is the neural activity changes with time, in which the blue area represents the period when AVBL was in high neuro activity, and the light green area represents the period when AVAL and AVEL were in high neuro activity. The other demonstrated the distribution of the neural activity changes. AVAL, Bin size = 20; AVBL, Bin size = 7.5; AVDL, Bin size = 5; AVEL, Bin size = 6; PVCL, Bin size = 5. (b) The neural activity of AVAR, AVBR, AVDR, AVER, and PVCR. The graph at the left of the panel is the neural activity changes with time, in which the blue area represents the period when AVBL was in high neuro activity, and the light green area represents the period when AVAL and AVEL were in high neuro activity. The other graphs demonstrated the distribution of the neural activity changes. AVAR, Bin size = 10; AVBR, Bin size = 20; AVDR, Bin size = 5; AVER, Bin size = 5; PVCR, Bin size = 5.

Supplementary references

1 Jarrell, T. A. *et al.* The Connectome of a Decision-Making Neural Network. *Science* **337**, 437 (2012).

2 Brittin, C. A., Cook, S. J., Hall, D. H., Emmons, S. W. & Cohen, N. Volumetric reconstruction of main Caenorhabditis elegans neuropil at two different time points. (2018).

3 Cook, S. J. *et al.* Whole-animal connectomes of both Caenorhabditis elegans sexes. *Nature* **571**, 63-71 (2019).

4 Brewer, J. C., Olson, A. C., Collins, K. M., Koelle, M. R. & Hart, A. C. Serotonin and neuropeptides are both released by the HSN command neuron to initiate C. elegans egg laying. *Plos Genetics* **15** (2019).

5 Ravi, B., Garcia, J. & Collins, K. The HSN egg-laying command neurons regulate the defecation motor program in Caenorhabditis elegans: Integration. (2019).

6 Franciszek, R. & Jan, K. Synaptic polarity of the command interneurons for Caenorhabditis Elegans directional motion. *Frontiers in Systems Neuroscience* **8**, 360-367 (2014).

7 Gray, J. M., Hill, J. J. & Bargmann, C. I. A circuit for navigation in Caenorhab - ditis elegans. *Proceedings of the National Academy of Sciences of the United States of America* **102**, 3184-3191 (2005).

8 Chalasani, S. H. *et al.* Dissecting a circuit for olfactory behaviour in Caenorhabditis elegans. *Nature* **450**, 63-70 (2007).

9 Lechner, M., Hasani, R., Zimmer, M., Henzinger, T. A. & Grosu, R. in *2019 International Conference on Robotics and Automation (ICRA).*

10 Hong, R. L., Riebesell, M., Bumbarger, D. J., Cook, S. J. & Sommer, R. J. Evolution of neuronal anatomy and circuitry in two highly divergent nematode species. *eLife Sciences* **8** (2019).

11 Nichols, A. L. A., Eichler, T., Latham, R. & Zimmer, M. A global brain state underlies C. elegans sleep behavior. *Science* **356**, 1247-1247 (2017).
